# Hepatitis B Virus Stimulated Fibronectin Facilitates Viral Maintenance and Replication through Two Distinct Mechanisms

**DOI:** 10.1371/journal.pone.0152721

**Published:** 2016-03-29

**Authors:** Sheng Ren, Jun Wang, Tie-Long Chen, Hao-Yu Li, Yu-Shun Wan, Nan-Fang Peng, Xi-En Gui, Ying Zhu

**Affiliations:** 1 The State Key Laboratory of Virology, College of Life Sciences, Wuhan University, Wuhan, China; 2 Tongji Medical College of Huazhong University of Science and Technology, Wuhan, China; 3 Department of Infectious Diseases, Zhongnan Hospital of Wuhan University, Wuhan, China; Indiana University, UNITED STATES

## Abstract

Fibronectin (FN) is a high molecular weight extracellular matrix protein that functions in cell adhesion, growth, migration, and embryonic development. However, little is known about the role of FN during viral infection. In the present study, we found significantly higher levels of FN in sera, and liver tissues from hepatitis B virus (HBV) patients relative to healthy individuals. HBV expression enhanced FN mRNA and protein levels in the hepatic cell lines Huh7 and HepG2. HBV infection of susceptible HepG2-sodium taurocholate co-transporting polypeptide cells also increased FN expression. We also found that transcriptional factor specificity protein 1 was involved in the induction of FN by HBV. Knockdown of FN expression significantly inhibited HBV DNA replication and protein synthesis through activating endogenous IFN-α production. In addition, FN interacted with the transforming growth factor β-activated protein kinase 1 (TAK1) and TAK1-binding protein complex and attenuated interferon signaling by inhibiting TAK1 phosphorylation. Furthermore, the nuclear translocation of NF-κB/p65 was found to be inhibited by FN. We also observed that FN promoted HBV enhancers to support HBV expression. These results suggest novel functions of endogenous FN involved in immune evasion and maintenance of HBV replication.

## Introduction

Hepatitis B virus (HBV) is a hepatotropic, non-cytopathic virus that can cause both acute and chronic infections of the liver and lead to development of hepatitis, cirrhosis, and hepatocellular carcinoma [1]. Approximately one-third of the world’s population has serological evidence of past or present infection with HBV, and 240 million people are chronic HBV surface antigen (HBsAg) carriers with 500,000 to 1.2 million deaths per year caused by HBV infections [2–4]. To date, the immunomodulator IFN-α is one of the few antiviral agents licensed for the treatment of chronic HBV infection[5].

Fibronectin (FN) exists as a protein dimer, consisting of two nearly identical polypeptide chains linked by a pair of C-terminal disulfide bonds [6]. Each FN monomer has a molecular weight of 230–250 kDa and contains three types of modules: type I, II, and III. Two types of FN are present in vertebrates: soluble plasma FN is a major protein component of blood plasma (approximately 300 μg/ml) and is produced in the liver by hepatocytes; insoluble cellular FN is a major component of the extracellular matrix that binds to the membrane-spanning receptor proteins, integrins. Similar to integrins, FN binds extracellular matrix components such as collagen, fibrin, and heparan sulfate proteoglycans. FN is secreted by various cells, primarily fibroblasts, as a soluble protein dimer and is then assembled into an insoluble matrix in a complex cell-mediated process. FN plays a major role in cell adhesion, growth, migration, and differentiation, as well as wound healing and embryonic development [7]. Altered FN expression, degradation, and organization has been associated with a number of pathologies, including cancer and fibrosis [8].

Type I IFN (mainly IFNα/β) are expressed in response to the stimulation of pattern recognition receptors produced by viral infection [9]. Depending on TLRs, the IFN signal pathway can be classified as TLR-dependent and TLR-independent signal pathway. In the TLR-dependent signal pathway, viral DNA was sensed by TLR9 and then recruits MyD88, IRAKs and activation of TRAF6, an ubiquitin E3 ligase. On the one hand, TRAF6 ubiquitination activates IRF7 mediated IFN production; on the other hand, TRAF6 interacts with TAK1-TAB complex to activate the phosphorylation of TAK1 and then induces the activation of the transcription factors NF-κB and AP-1 to induce type I IFN production, respectively[10–12].

Previous studies have indicated that FN can facilitate the entry of gammaretrovirus, influenza A virus, and rhabdovirus [13–15]. Regarding HBV, FN was found to bind HBsAg [16, 17], is capable of accelerating HBV infection in primary cultured fetal hepatocytes [18], and may be important for HBV propagation [19]. In addition, HBV X antigen up-regulates FN expression and is co-expressed with FN mRNA [20]. All these studies imply that FN may play a role during HBV replication and expression, but the detailed molecular mechanisms by which FN affects HBV replication remain unclear.

In this study, we focused on the mechanisms underlying the essential role of FN in HBV replication and expression and identified two previously unrecognized functions of endogenous FN during HBV replication. Firstly, HBV upregulated the expression of FN in both clinical samples and cultured cells. In turn, the increased FN antagonized the endogenous interferon pathway, leading to enhanced HBV DNA replication and protein synthesis. Secondly, FN promoted HBV replication and expression by strengthening the recruitment of hepatocyte nuclear factor 4 alpha (HNF-4α) to HBV enhancer II (EII). Our results reveal the importance of liver-specific host factors that contribute to HBV maintenance.

## Materials and Methods

### Clinical Samples and Human Subjects

Peripheral blood samples were obtained from 50 patients with chronic hepatitis B (CHB) admitted to Zhongnan Hospital, Wuhan University (33 males and 17 females, with a mean age of 48.1±11.6 years). Matched for sex and age, 50 healthy individuals (27 males and 23 females with a mean age of 43.5±10.5 years) with no history of liver disease and negative for virus infection were randomly selected as controls from the local blood donation center. Liver tissues were obtained from 15 patients with CHB admitted to Zhongnan Hospital, Wuhan University (10 males and 5 females with a mean age of 52.1±12.9 years). Matched for sex and age, 15 healthy individuals (12 males and 3 females with a mean age of 49.1±8.39 years) negative for virus infection were randomly selected as controls. All clinical samples were collected from September 6, 2014 to September 23, 2014. Whole study (including the collection of blood and tissue sample) was conducted according to the principles of the Declaration of Helsinki and approved by the Institutional Review Board of the College of Life Sciences, Wuhan University, in accordance with its guidelines for the protection of human subjects. Written informed consent was obtained from each participant in this study.

### Cell Culture and Cell Line Construction

The HEK293 (CRL-11268) and human hepatoma cell lines HepG2 (HB-8065) were purchased from ATCC and grown in DMEM supplemented with 10% heat-inactivated FBS, 100 U/ml penicillin, and 100 U/ml streptomycin sulfate at 37˚C in 5% CO_2_. The Huh7 and the HepG2.2.15 cell line, which was derived from HepG2 cells and stably expresses HBV (subtype ayw), were provided by Prof. Jian-Guo Wu (Wuhan University, Wuhan, China) and has been described in a previous study [21]. The Huh7.3.7 cell line, which was derived from Huh7 cells and also stably expresses HBV (subtype adw), has been constructed in our lab and described in previous studies [22, 23]. The stable cell lines were maintained in DMEM containing 500 mg/ml G418. Below is a brief protocol for constructing a stable FN knockdown cell line; detailed information is available at http://www.addgene.org/tools/protocols/plko/. HEK293 cells were transfected with shFN-1 or shNC along with pMD2.G and psPAX2. The supernatants of the transfected cells were then collected and filtered by a 0.45 μm filter to harvest the lentivirus. The FN stable knockdown cell line (FN-KD) and control stable knockdown cell line (NC-KD) were obtained by puromycin pressure screening in Huh7 cells, which were transduced with the packaged lentiviruses. The knockdown cell lines were maintained in DMEM containing 2.5 μg/ml puromycin. HEK293 cells were transfected with pFB-hNTCP along with pMD2.G and psPAX2. The supernatants of the transfected cells were then collected and filtered by a 0.45 μm filter to harvest the lentivirus. The HepG2-hNTCP cell lines were obtained by G418 pressure screening in HepG2 cells transduced with the packaged lentiviruses. The HepG2-hNTCP cell lines were maintained in DMEM containing 500 mg/ml G418.

### Analysis of HBV Core-Associated DNA by Quantitative PCR (qPCR)

HBV core-associated DNA, which is found in HBV core particles inside transfected cells, was analyzed by qPCR. The primer sequences and the probe used in qPCR are shown in S3 Table. The detailed information is described in previous studies [22, 24].

### HBV Infection of HepG2-hNTCP Cells

HepG2-hNTCP cells were cultured in primary hepatocytes maintenance medium (PMM) for 24 h and then inoculated with 200 multiplicity of genome equivalents of HBV in PMM with 5% PEG 8000 at 37°C for approximately 24 h. One day after infection, cell were washed with PBS three times to remove residual viral particles and maintained in the same medium containing 2.5% DMSO. The medium was refreshed every other day.

### SeV Infection

Cells were seeded in proper dishes and cultured overnight, and then cells were incubated with 10% FBS DMEM containing SeV (MOI = 1) or equivalent PBS for indicated time.

### Plasmids and Reagents

Coding regions of FN were generated by PCR amplification. The PCR products were digested with NheI/HindIII and cloned directly into the pCDNA-3.1(+) expression vector to generate full-length pFN. Full length or truncated domains of FN was subcloned to PKH3-3XHA. TAK1, TAB1, IκBα and NEMO were cloned into p3XFlag-CMV-14. TAB2, TAB3 were cloned into pcDNA3.1-Myc. Specific short hairpin RNA (shRNA) against FN or off-target control (shFN-1, shFN-2, shOF-1, and shOF-2) or negative control (shNC) were constructed according to the methods on the website http://www.addgene.org/tools/protocols/plko/, based on the sequence in S3 Table. Plasmids pMD2.G and psPAX2 for producing lentivirus were purchased from Addgene. The pHBV (ayw) was generated from the HBV genome (genotype D, subtype ayw, GenBank accession no. U95551), digested with EcoRI/ SalI, and inserted into pBluescript II; pHBV (adw) was generated from the HBV genome (genotype B, subtype adw, GenBank accession no. JN406371, http://www.ncbi.nlm.nih.gov/genbank/), digested with EcoRI/ SalI, and inserted into pBluescript II. WT promoters of FN, IFN-α and enhancers of HBV were cloned to pGL3-Basic, various mutation were subcloned according to the sequence in S3 Table. All constructs were verified by sequencing. Antibodies against FN (15613-1-AP, 66042-1-Ig), HA-tag (51064-2-AP), Sp1 (21962-1-AP), GAPDH (60004-1-Ig), GFP (66002-1-Ig) and β-actin (60008-1-Ig) were purchased from Proteintech Group (Wuhan, China). Antibodies against HBV core (sc-23947), HBV surface (sc-57762), PKR (sc-6282), OAS2 (sc-271117), MxA (sc-271399), Myc-tag (sc-42), TAB1 (sc-6053), TAB2 (sc-11851), c-Jun (sc-44), HNF-4α (sc-8987), TAK1 (sc-166562), p-TAK1 (sc-130219), p50 (sc-53744), p65 (sc-372) and Lamin A (sc-6214) were purchased from Santa Cruz Biotechnology (USA). TAB3 (ab124723) antibody was purchased from Abcam (Cambridge, UK), and Flag-tag antibody (637301) from BioLegend (USA). NF-κB inhibitor, BAY-11-7082 was purchased from Sigma (St. Louis, MO).

### Reverse-Transcription Reaction and Quantitative Real-Time PCR (qRT-PCR)

Total RNA was isolated with TRIzol (Invitrogen, Carlsbad, CA). Cellular RNA samples were reverse transcribed with random primers. The qRT-PCR was performed with a Light Cycler 480 (Roche, Indianapolis, IN). GAPDH or actin was amplified as an internal control, and the primer sequences are shown in S3 Table.

### Transfection and Luciferase Reporter Gene Assays

The detailed information is described in previous studies [22, 24]. Assays were performed in triplicate, and the results were expressed as mean percentage ± SD relative to the vector or mock control samples, which were set as 1.

### Western Blot Analysis

The detailed information is described in previous studies[22].

### ELISA Quantification of HBV e Antigen (HBVeAg), HBVsAg, and FN

Cells were transfected as indicated and cultured for an additional 48 h in DMEM without FBS or antibiotics. The conditioned media were collected, and a standard ELISA kit was used to quantify HBeAg and HBsAg (Shanghai KeHua Biotech, Shanghai, China). Serum was diluted and measured according to a standard ELISA kit for FN (eBioscience, Shanghai, China).

### Histology and Immunohistochemistry

Immunohistochemical analysis of FN expression was performed as previously described[25].

### Immunoprecipitation

Immunoprecipitation analysis of was performed as previously described [26]

### ChIP

The detailed information is described in previous studies [23]. Primers for PCR amplification the FN promoter (containing two sp1 binding site) or HBV enhancers are shown in S3 Table.

### Statistical Analysis

FN protein levels in serum represent the means ± SEM. The relationships between FN protein expression and HBV DNA in patient samples were analyzed by Pearson’s correlation. All experiments (except clinical samples) were reproducible and carried out in triplicate. Each set of experiments was repeated at least three times with similar results and a representative of similar results was shown. The results are presented as the mean ± SD. Two-tailed Student’s t-test for paired samples was used to determine statistical significance. Differences were considered statistically significant at the following P values: **P<0.01, *P<0.05.

## Results

### FN Expression Is Elevated in HBV Patients

**Fig 1:**
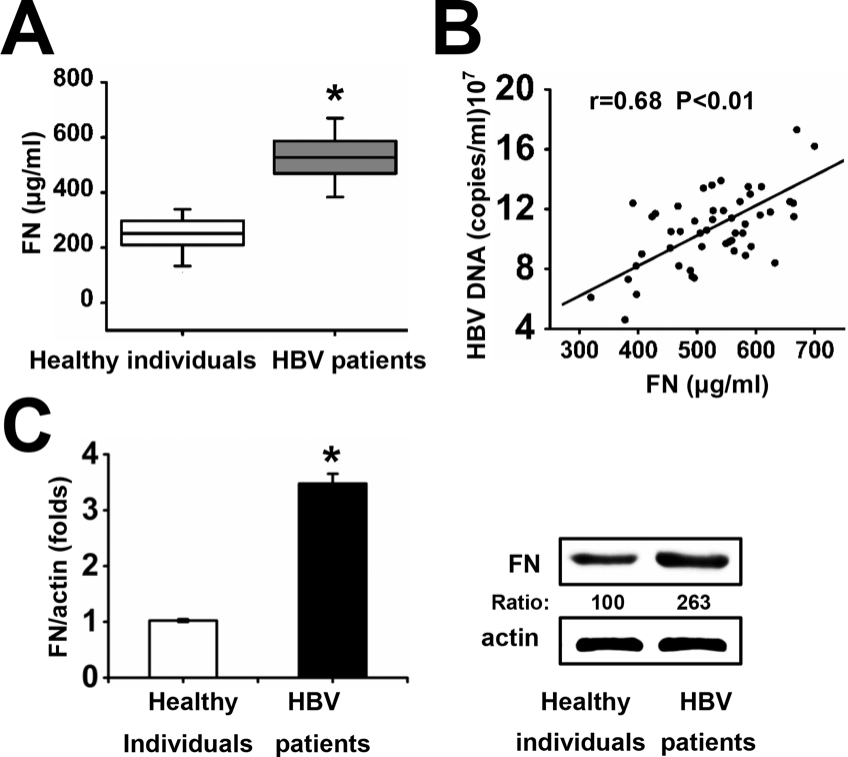
FN expression in healthy individuals and patients infected with HBV. (A) Serum FN protein levels in healthy individuals (n = 50) and HBV patients (n = 50) were measured by ELISA. Data represent mean ± SEM (*p<0.05). (B) Correlation analysis between FN protein levels and HBV DNA loads in serum from CHB patients (n = 50). Solid line, linear growth trend; r, correlation coefficient; Student’s *t*-test was used to determine *P* values. (C) FN expression was detected by qRT-PCR and western blots with normal liver biopsies (n = 15) and liver biopsies with CHB (n = 15). Data represent means ± SD, n = 3 (*p<0.05). Numbers below the blots are the quantified optical density; control blots were set as 100.One representative result is shown in each group.

In order to investigate FN expression during HBV infection, serum FN protein levels in HBV patients were determined by ELISA. The serum levels of FN were significantly higher in patients with CHB infection (n = 50) than in healthy individuals (n = 50) (mean ± SEM: 531.69 ± 94.75 vs. 250.21 ± 59.64 μg/ml) (Fig 1A, S1 Table), suggesting that HBV infection resulted in the up-regulation of circulating FN.

In order to investigate whether active viral replication correlated with FN expression, we analyzed the correlation between FN expression and the clinical virological characteristics of CHB patients. There was a linear positive correlation between HBV DNA level and FN expression in serum from CHB patients (Fig 1B, S1 Table).

Furthermore, FN mRNA and protein levels in liver tissue were detected by qRT-PCR and western blots. Similarly, higher expression of FN were observed in HBV patients than healthy individuals (Fig 1C, S2 Table). Together, these data indicate that FN expression is elevated in HBV patients.

### FN Expression Is Increased by HBV in Various Cell Types

**Fig 2:**
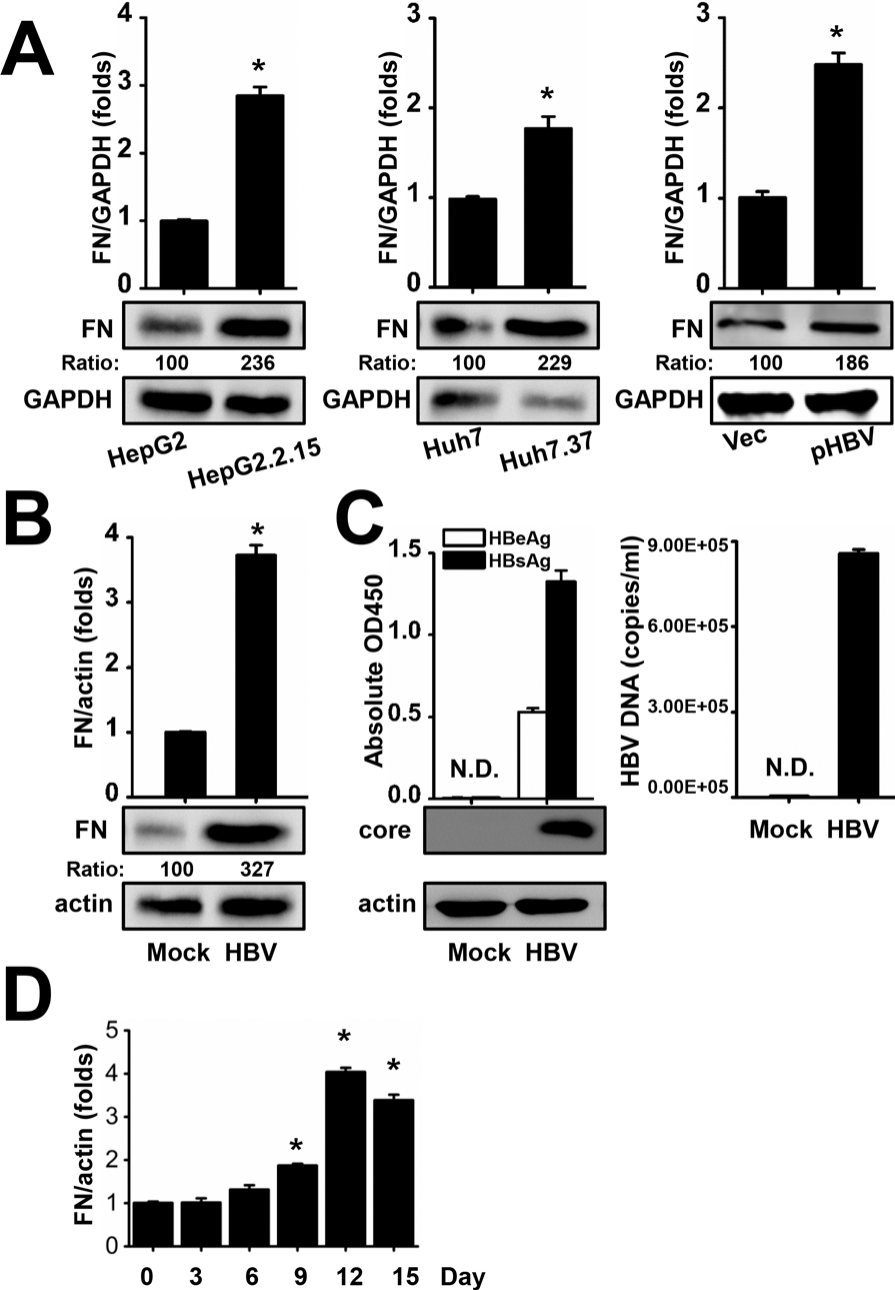
Effect of HBV on FN expression. (A) The indicated cells were serum starved for 24 h and then FN expression was determined by qRT-PCR and western blot (left and middle panel). Huh7 cells were transfected with vector or pHBV (ayw) and 48 h later, FN expression was determined by qRT-PCR and western blot (right panel). (B) Mock-infected or HBV-infected HepG2-hNTCP cells were analyzed for FN expression 11 days post-infection. (C) HBe, HBs, HBV core protein, and HBV DNA were detected 11 days after infection. N.D., not detected. (D) HBV-infected HepG2-hNTCP cells were analyzed for FN expression at indicated days post-infection. Numbers below the blots are the quantified optical density; control blots were set as 100. All experiments were repeated at least three times with similar results. Data represent means ± SD, n = 3 (*p<0.05).

High levels of FN expression were detected in HBV patients, so we next determined whether or not endogenous FN expression in cell culture was influenced by HBV. FN mRNA and protein levels were determined in HepG2 human hepatic cells and HepG2.2.15 cells, which contain an integrated HBV (subtype ayw) genome and stably express HBV [21]. We found that FN mRNA and protein expression in HepG2.2.15 cells was significantly elevated compared with HepG2 cells (Fig 2A, left panel). In addition, Huh7 human hepatic cells and Huh7.37 cells, another HBV-positive stable cell line (subtype adw) [22, 23], were examined for FN expression and similar results were observed (Fig 2A, middle panel). Huh7 cells were then transfected with empty vector or pHBV, a plasmid containing 1.3-fold over length fragment of the HBV genome (subtype ayw) that retains the ability to produce mature HBV virions. The expression levels of FN mRNA and protein were increased by HBV (Fig 2A, right panel).

As NTCP was identified as a functional receptor for HBV [27], we constructed a HepG2-hNTCP stable cell line and investigated the effect of HBV infection on FN expression. Indeed, we found that infection of HepG2-hNTCP cells with HBV increased FN expression (Fig 2B). In order to ensure that the infection was successful, the HBV core protein, HBeAg, HBsAg, and DNA levels were determined (Fig 2C). In addition, the kinetic of FN expression after HBV infection in HepG2-hNTCP cells was measured and significant induction of FN was observed on day 9 (Fig 2D). Together, the results indicate that HBV stimulated FN expression at both the mRNA and protein level in hepatocytes.

### FN Transcription Was Induced by HBV Primarily through Sp1 Activation

**Fig 3:**
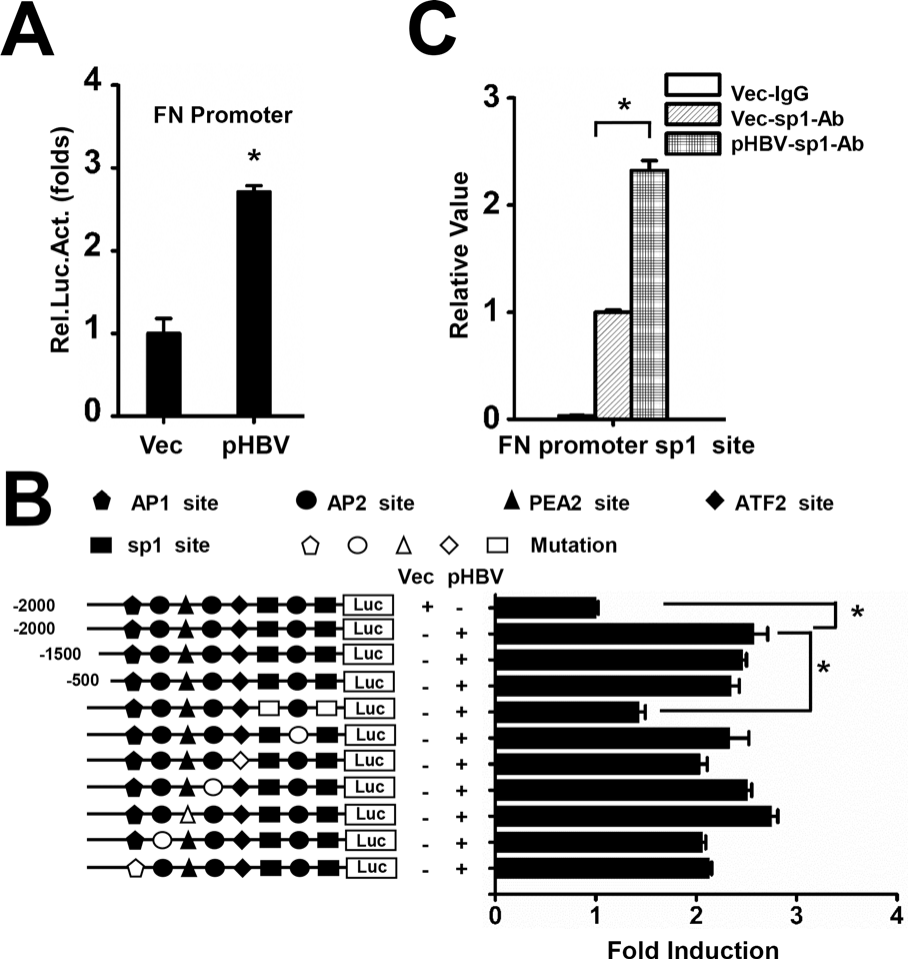
HBV promotes FN expression by activating Sp1 activities. (A) Huh7 cells were transfected with luciferase reporter plasmids containing the FN promoter (pFN-promoter-Luc) along with pHBV or vector. Cell culture medium was refreshed with serum-free medium 6 h post-transfection. Luciferase activity was measured 24 h after serum starvation. (B) Huh7 cells were transfected with wild-type, truncated, or mutated FN promoters along with pHBV and then cell culture medium was refreshed with serum-free medium 6 h post-transfection. Luciferase activity was measured 48 h after serum starvation. (C) Huh7 cells were transfected with empty vector or pHBV and then ChIP analysis was performed to assess Sp1 binding capability to FN promoter 48 h post-transfection. All experiments were repeated at least three times with similar results. Data represent means ± SD, n = 3 (*p<0.05).

As we found that HBV increased FN expression at both clinical and cellular stage, it is naturally to uncover the mechanism behind it. The pFN-promoter-Luc was co-transfected along with pHBV-1.3 into Huh7 cells to determine where FN expression was being influenced by HBV. Luciferase activity was measured in each sample, and we found that HBV activated FN promoter activities (Fig 3A), implying that FN was induced by HBV at a transcriptional level.

Several transcriptional factors such as activating protein 1 (AP1), AP2, polyomavirus enhancer A binding protein 2 (PEA2), activating transcription factor 2 (ATF2), and Sp1 are important regulatory elements within the FN promoter [28–30]. In order to identify the cis-regulatory elements in the FN promoter that are responsive to HBV, truncation reporter plasmids and mutant reporter plasmids were constructed, as the binding sites of these factors were mutated in the FN promoter. We found that only mutation of Sp1 binding sites had significant effects on the induction of the FN promoter by HBV (Fig 3B), whereas AP1, AP2 and ATF2 also have mild influence. ChIP assays were then performed to test the Sp1 binding activity on the corresponding region within the FN promoter. The results showed that binding of Sp1 on the FN promoter was significantly strengthened by HBV (Fig 3C). Together, these results suggest that HBV stimulated FN transcription by activating Sp1.

### FN Facilitates HBV Replication and Expression

**Fig 4:**
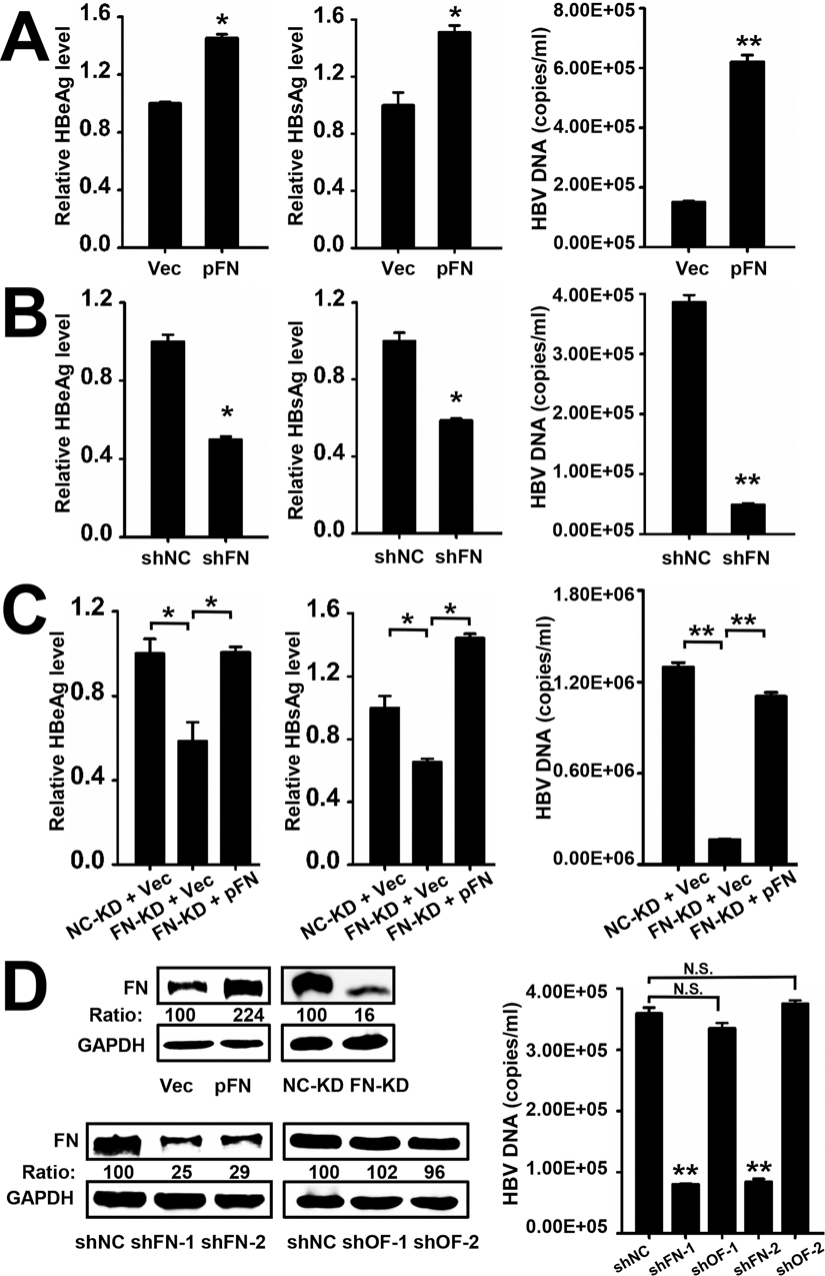
FN enhances the replication and expression of HBV. HepG2.2.15 cells were transfected with pFN (A) or shFN (B) and then 48 h after transfection, the supernatants were collected and assayed for HBeAg and HBsAg by ELISA. HBV core-associated DNA production was analyzed by qPCR. An empty vector or an irrelevant shRNA was used as a control. (C) NC-KD or FN-KD cells were transfected with empty vector or pFN along with pHBV. At 48 h after transfection, the supernatants were collected and assayed for HBeAg and HBsAg by ELISA, and HBV core-associated DNA production was analyzed by qPCR. (D) Huh7 cells were transfected with indicated plasmids and 48 h later, FN expression was determined by western blot. NC-KD and FN-KD were serum starved for 24 h and then FN expression was determined by western blot. Huh7 cells were transfected with shNC, shFN or seed matched FN off-target shRNA (shOF) along with pHBV. At 48 h after transfection, HBV core-associated DNA production was analyzed by qPCR. Numbers below the blots are the quantified optical density; control blots were set as 100. All experiments were repeated at least three times with similar results. Data represent means ± SD, n = 3 (**p<0.01; *p<0.05).

As HBV stimulated FN production, we next explored the biological function of FN during HBV replication. HepG2.2.15 cells were transfected with pFN, and we found that over-expression of FN stimulated HBeAg and HBsAg expression and enhanced HBV DNA replication (Fig 4A). In contrast, knockdown of FN by specific shRNA, shFN-1, suppressed HBeAg, HBsAg, and HBV DNA levels (Fig 4B). Similar results were observed with another shFN, shFN-2.

A stable FN knockdown cell line (FN-KD) and a negative control knockdown stable cell line (NC-KD) were constructed to confirm the effect of FN on HBV replication using a lentiviral vector expressing FN-specific shRNA or irrelevant shRNA. We then performed MTT assays to measure cell viability to determine whether or not the changes in FN expression affected cell growth. As expected, over-expression or knockdown of FN did not significantly change Huh7 cell viability (S1 Fig). We next sought to examine HBV replication and expression in stable FN knockdown cells. Consistent with the above results, significantly lower levels of HBeAg, HBsAg, and HBV DNA were detected in stable FN knockdown cells compared with control cells. In addition, HBV expression and replication were rescued when FN was restored by transfection of pFN into stable FN knockdown cells (Fig 4C). The knockdown and over-expression efficiency of FN were determined by western blot (Fig 4D). Our results showed that the knockdown of FN has more significant effect on viral replication than the over-expression of FN due to relatively high basal FN expression in hepatocytes. In order to avoid off-target effects [31], seed sequence-matched controls were designed to detect FN depletion and the influences on HBV expression and replication, and we found that these controls did not reduce FN or HBV expression (Fig 4D). To further clarify the increase of HBV antigen levels in culture medium is come from the elevated expression of viral genes or the enhanced efficiency of antigen secretion, the intracellular HBsAg level was determined and variation was consistent with the secreted HBsAg and HBV DNA level (S2 Fig). So it is clear that FN enhance the viral genes expression. Taken together, these results demonstrate that HBV expression and replication is FN dependent.

### FN Inhibits the Expression of IFN-α and IFN-Stimulated Genes

**Fig 5:**
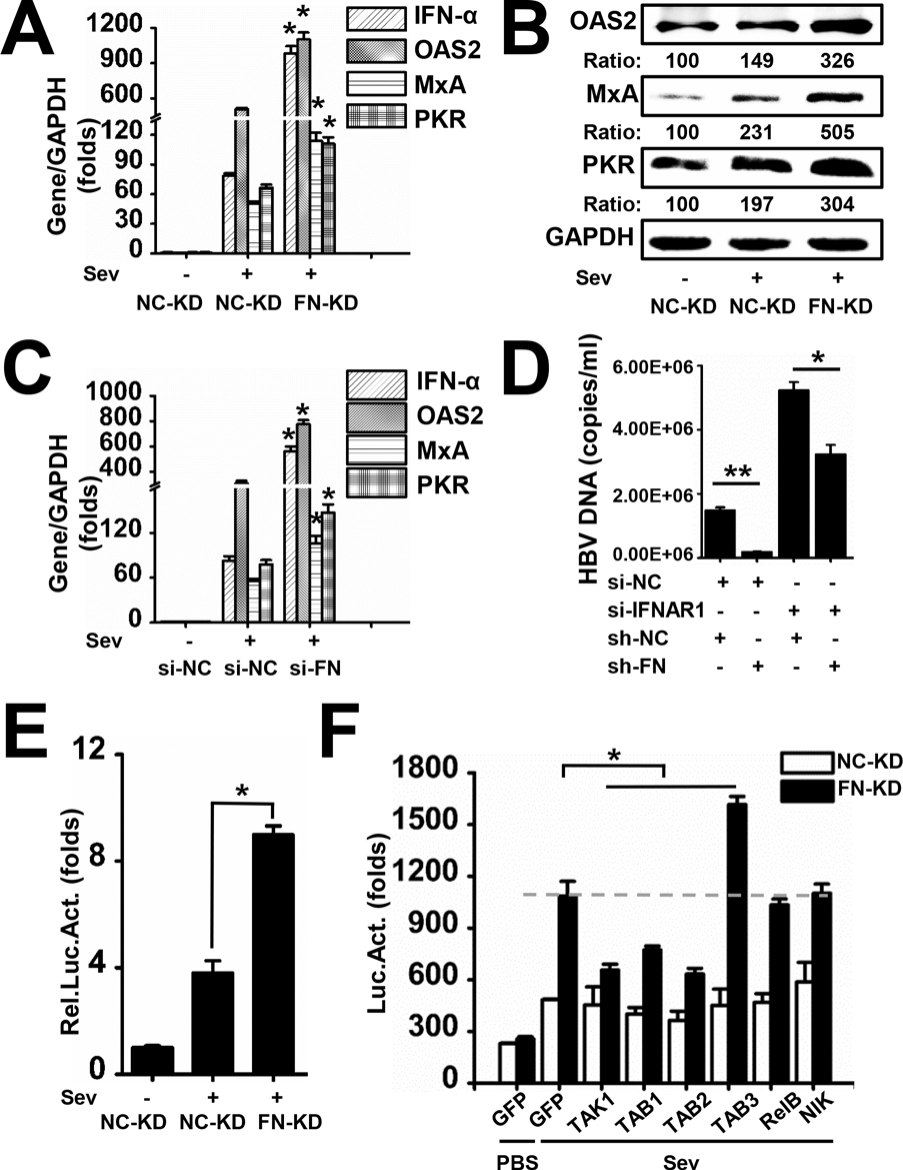
FN inhibits IFN-α expression induced by viral infection. (A) NC-KD cells and FN-KD cells were stimulated with SeV (MOI = 1) or PBS for 48 h, and then expression of IFN-α, OAS2, MxA, and PKR were determined by qRT-PCR. (B) NC-KD and FN-KD cells were stimulated with SeV (MOI = 1) or PBS for 48 h, and then expression of OAS2, MxA, and PKR was determined by western blot. (C) NC-KD cells were treated with si-NC or si-FN for 24 h and followed by stimulation with SeV (MOI = 1) or PBS for 48 h, and then expression of IFN-α, OAS2, MxA, and PKR were determined by qRT-PCR. (D) NC-KD cells were treated with indicated si-RNA and/or sh-RNA for 24 h and then transfected with pHBV (ayw) for 48 h. The production of HBV core-associated DNA was analyzed by qPCR. (E) NC-KD and FN-KD cells were transfected with luciferase reporter plasmids containing the IFN-α2 promoter, cells were stimulated with SeV (MOI = 1) or PBS for 24 h, and then luciferase activity was measured. (F) NC-KD and FN-KD cells were transfected with GFP vector or the constructs expressing the indicated genes along with the IFN-α2 promoter, cells were treated with SeV (MOI = 1) or PBS for 24 h, and then luciferase activity was measured. Numbers below the blots are the quantified optical density; control blots were set as 100. All experiments were repeated at least three times with similar results. Data represent means ± SD, n = 3 (*p<0.05).

As our above results suggest that FN enhances HBV replication and expression, we then set out to determine the mechanisms behind this phenomenon. IFN-α is an antiviral agents used for the treatment of chronic HBV infection [32, 33], so we determined whether FN could influence production of endogenous IFN-α. NC-KD and FN-KD cells were mock infected or Sendai virus (SeV, MOI = 1) infected and then expression of IFN-α and IFN-stimulated genes (ISGs), the direct antiviral effectors such as oligoadenylate synthetase (OAS) 2, double-stranded RNA-dependent protein kinase (PKR), myxovirus (influenza) resistance A protein (MxA), were measured by qRT-PCR. We found that IFN-α, OAS2, MxA, and PKR mRNA levels were induced dramatically in FN-KD cells compared with NC-KD cells (Fig 5A). In addition, protein expression of OAS2, MxA, and PKR were also significantly higher in FN-KD cells than in NC-KD cells (Fig 5B). In order to confirm that the results are valid and not due to a selective event during the selection of the stable cell lines, NC-KD cells were treated with si-FN or si-NC and followed by SeV or mock infection. And similar results were observed as in stable knockdown cells (Fig 5C). To prove that it is FN-mediated down-regulation of IFN-α and ISGs that are responsible for the increase of HBV, HBV DNA was detected and compared in the presence or absence of IFN. The inhibition effect on HBV DNA by FN depletion was decrease by 40% in the presence of siRNA-IFNAR1 contrasted with 90% in the siRNA-NC group (Fig 5D). Similar results were observed when luciferase reporter assays were performed for IFN-α2 promoter activities (Fig 5E). These data demonstrate that FN indeed has an inhibitory effect on the production of endogenous IFN-α and downstream ISGs.

In order to determine if there was crosstalk between FN and the IFN-α signaling pathway, NC-KD cells and FN-KD cells were transfected with pIFN-α2-Luc, which carries an IFN-α2 subtype promoter, along with the indicated constructs expressing genes involved in endogenous IFN-α signaling, followed by mock infection or SeV infection. We found that TAK1 and TAB1, 2, and 3, but not RelB or NF-κB-inducing kinase (NIK), significantly changed the effect of FN on IFN-α2 promoter activities (Fig 5F). These data imply that the TAK1-TAB complex may be involved in FN-mediated IFN-α expression.

### FN Interacts with the TAK1-TAB Complex and Inhibits Phosphorylation of TAK1

**Fig 6:**
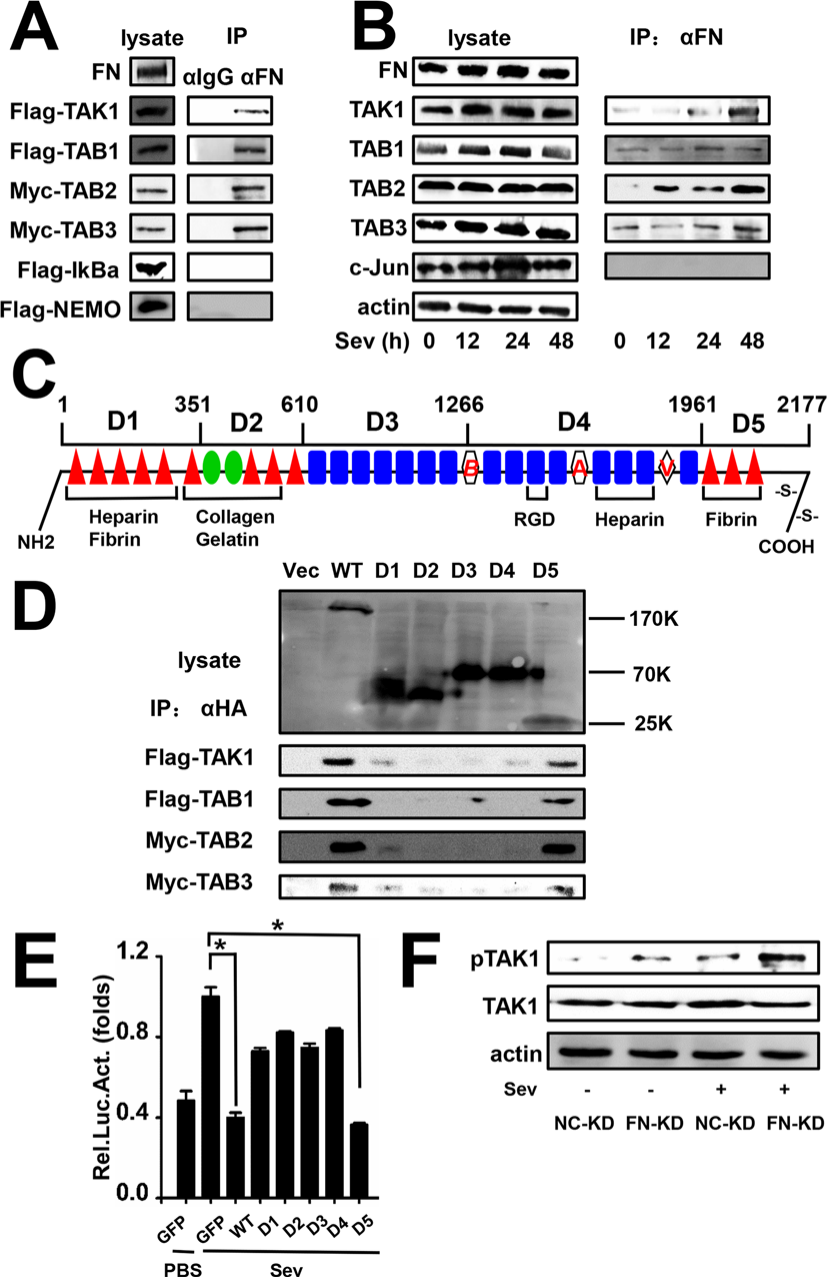
FN interacts with the TAK1-TAB1, 2, and 3 complexes. (A) HEK293 cells were transfected with TAK1, TAB1, TAB2, TAB3, IkBα, or NEMO with indicated tags along with pFN. At 48 h post-transfection, Co-IP and immunoblot analysis was performed with the indicated antibodies. The expression levels of transfected FN, TAK1, TAB1, TAB2, TAB3, IkBα, and NEMO were detected by western blot. (B) Huh7 cells were stimulated with SeV (MOI = 1) at the indicated times. Co-IP and immunoblot analysis was performed with the indicated antibodies. The expression levels of endogenous FN, TAK1, TAB1, TAB2, TAB3, and c-Jun were detected by western blot. (C) A schematic diagram of the functional domains of FN and the five truncated segments. The number shows the amino acid sequence of each segments. (D) HEK293 cells were transfected with empty vector or full length HA-FN or each of five HA-FN-truncated constructs, D1–5 along with Flag-TAK1, Flag-TAB1, Myc-TAB2, or Myc-TAB3. At 48 h post-transfection, Co-IP and immunoblot analysis was performed with the indicated antibodies. (E) Constructs expressing GFP, wild-type FN or each of five truncated FN D1–5 were transfected along with IFN-α2 promoter and cells were stimulated with SeV (MOI = 1) or PBS. At 24 h post-transfection, cells were harvested to measure luciferase activity. (F) NC-KD and FN-KD cells were stimulated with SeV (MOI = 1) or PBS for 48 h and TAK1 and p-TAK1 expression was determined by western blot. All experiments were repeated at least three times with similar results. Data represent means ± SD, n = 3 (*p<0.05).

Based on the above results, we inferred that FN may crosstalk with the IFN pathway through association with the TAK1-TAB complex. TAK1, TAB1, TAB2, TAB3, IkBα, and NF-κB essential modulator (NEMO) with the indicated tags were transfected into HEK293 cells along with pFN. Cells were harvested for Co-IP assays. We found that exogenous FN interacts with the TAK1-TAB complex including TAK1, TAB1, TAB2, and TAB3, but not IkBα or NEMO (Fig 6A). Furthermore, we performed Co-IP assays to detect the interaction between endogenous FN and TAK-TAB. As shown in Fig 6B, their interactions increased after SeV infection.

As the schematic diagram of the FN protein structure reveals (Fig 6C) [34, 35], FN has many repeated domains primarily classified as module I, module II, and module III. Additional domains A, B, and variable region (V) only exist in infants or in some special tissues and conditions [36, 37]. In order to determine which FN domain associates with the TAK1-TAB complex, five truncated segments of FN (D1–D5) were constructed as indicated in the sketch map (Fig 6C). Co-IP assays were performed to determine the association between each of the five FN segments (D1–D5) and the TAK1-TAB complex. We found that the FN WT and D5 was associated with TAK1, TAB1, TAB2, and TAB3 and that there was very little association between D1,D2,D3,D4 and the TAK1-TAB complex (Fig 6D).

In order to confirm that D5 was a functional domain, a GFP control, wild-type FN, or each of the five FN mutants were transfected along with the IFN-α2 promoter into HEK293 cells, followed by mock infection or SeV infection. Cells were then harvested and luciferase activity measured. The results showed that IFN-α2 promoter activities were significantly inhibited by WT FN or D5 but only moderately affected by D1, D2, D3, or D4 (Fig 6E). Taken together, these results suggest that FN is indeed associated with the TAK1-TAB complex and the main binding part located at the C-terminal domain, D5. We then determined the effect of FN on TAK1 phosphorylation to further explore the biological significance of the above results. As Fig 6F shows, TAK1 phosphorylation induced by viral infection was significantly enhanced by the depletion of FN.

As FN interacts with both HBsAg and the TAK1-TAB complex, there may be competition between HBsAg and TAB3 for the same FN binding site. In order to clarify this point, GFP control or Flag-HBV large surface protein (HBL) was co-transfected along with pFN and myc-TAB3 into HEK293 cells. Co-IP assays were then performed to determine protein interactions. We found that HBL strengthens the association between FN and TAB3 (S3 Fig), indicating that there was no competition between HBsAg and TAB3.

Current knowledge of FN are focused on the extracellular type. The role of intracellular FN remains largely unknown. Whether the extracellular FN interacts with TAK1-TAB complex indirectly through transmembrane protein or the intracellular FN interacts with TAK1-TAB complex directly needs to be clarified. In order to confirm whether the intracellular FN exists, Huh7 cells were thoroughly digested with trypsin proteinase to degrade the extracellular FN[38–40], the cells were then examined for the expression of FN. As S4A Fig shows, there was surely intracellular FN existence in the cells although the expression level is lower than the extracellular FN. We also discovered that the FN-TAK1 interaction increased in the presence of HBV expression (S4B Fig) which is consistent with the results in Fig 6B. Furthermore, we used THP-1, a suspension cell line without extracellular matrix, to get another evidence to prove that the intracellular FN interacts with TAK1 (S4C Fig).

### NF-κB Is Involved in the Inhibition of IFN-α Expression by FN

**Fig 7:**
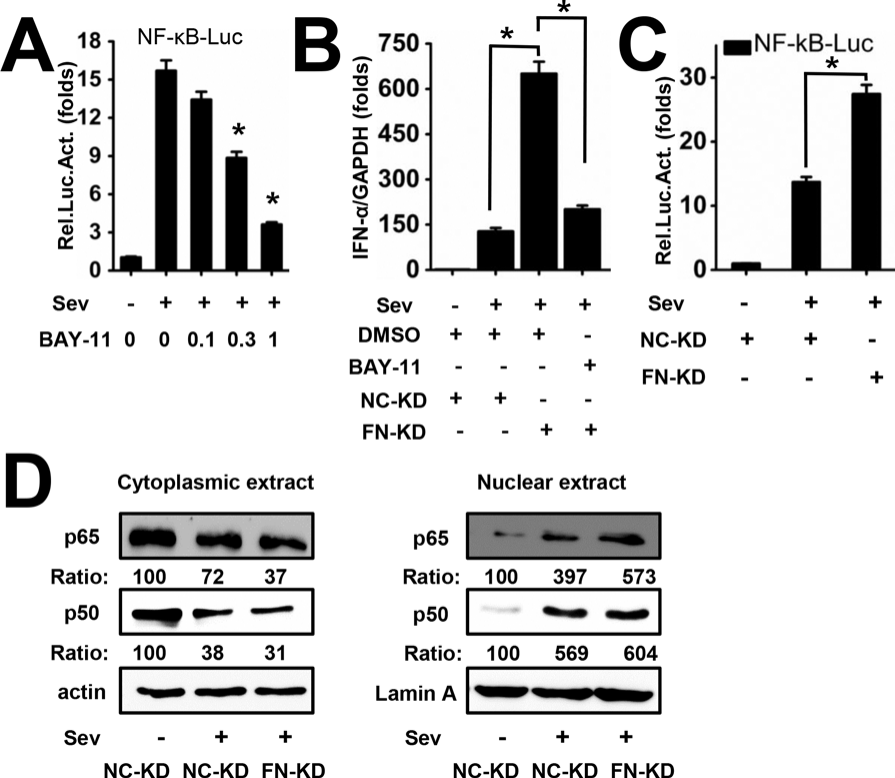
NF-κB is involved in the inhibition of IFN-α by FN. (A) NC-KD cells were transfected with pNF-κB-Luc (5 ×NF-κB binding site promoter driven luciferase reporter plasmid). At 24 h post transfection, cells were mock infected or SeV (MOI = 1) infected along with the treatment of indicated concentration of BAY-11 (μM). Cells were harvested for luciferase assay at 48 h post transfection. (B) NC-KD or FN-KD cells were mock infected or Sev (MOI = 1) infected. At 24 h post infection, cell culture were replaced with medium containing DMSO or BAY-11 (1 μM) for another 24 h. IFN-α mRNA level was determined by qRT-PCR. (C) NC-KD and FN-KD cells were transfected with pNF-κB-Luc. At 24 h post transfection, cells were mock infected or SeV (MOI = 1) infected. Cells were harvested for luciferase assay at 48 h post transfection.(D) NC-KD and FN-KD cells were mock infected or SeV (MOI = 1) infected for 12 h. Cytoplasmic and nuclear fractions were prepared for western blot with indicated antibodies to determine the localization of NF-κB subunits. All experiments were repeated at least three times with similar results. Data represent means ± SD, n = 3 (*p<0.05).

As NF-κB is the key regulator downstream the TAK1-TAB complex [12], we next explore whether NF-κB mediates the suppression of IFN-α by FN. First, the inhibition effect on NF-κB pathway by BAY-11, an NF-κB inhibitor, was detected (Fig 7A). And then the results showed that IFN-α activation by the knockdown of FN was suppressed by BAY-11 (Fig 7B), suggesting that NF-κB in involved in the inhibition of IFN-α expression by FN. In addition, the effect of FN on NF-κB activity was investigated using a luciferase reporter assay. The result in showed that FN significantly suppressed the activity of NF-κB elicited by Sev stimulation in Huh7 cells (Fig 7C). Furthermore, the nuclear translocation of NF-κB/p65 but not p50 was found to be inhibited by FN (Fig 7D). Taken together, FN inhibits IFN-α production through decreasing the nuclear transport of NF-κB/p65.

**Fig 8:**
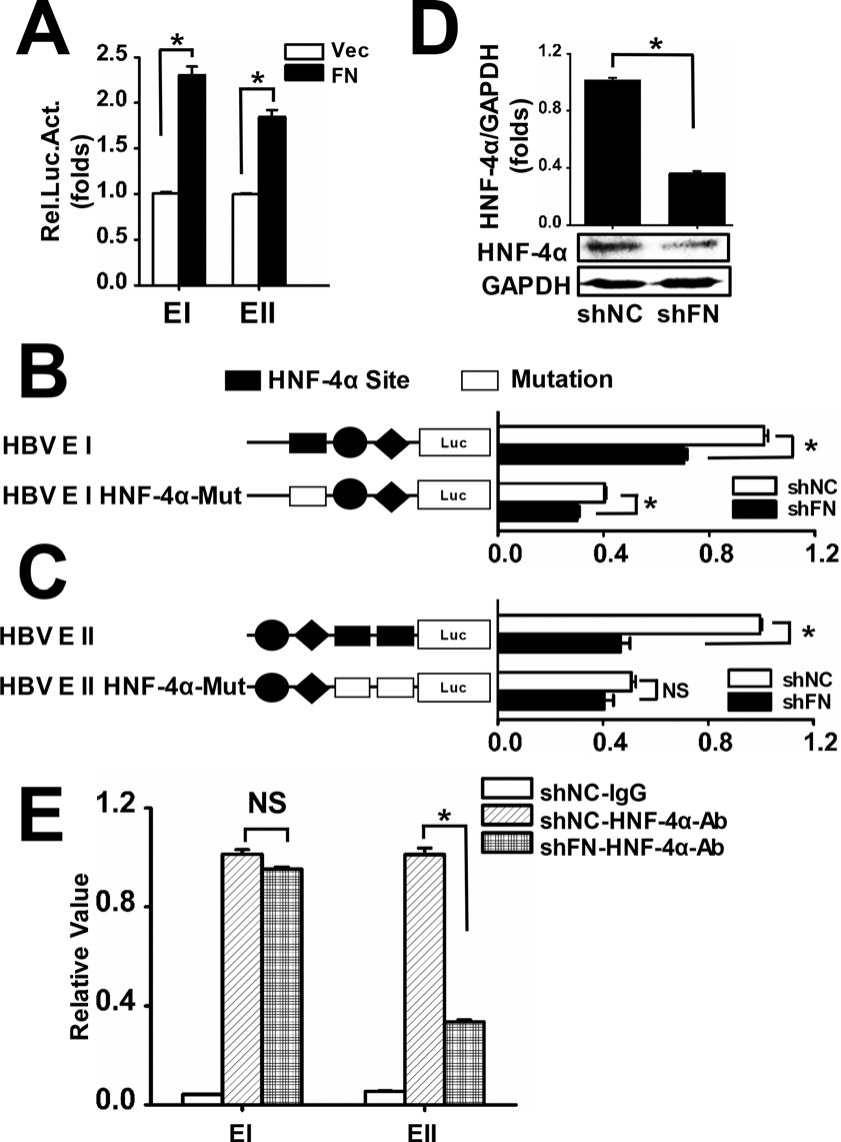
FN promotes HBV expression by activating HBV EI and EII activities. (A) Huh7 cells were transfected with HBV EI or EII along with pFN and then cell culture medium was refreshed with serum-free medium 6 h post-transfection. Luciferase activity was measured 24 h after serum starvation. Huh7 cells were transfected with wild-type EI or mutant EI HNF-4α-Mut (B), wild-type EII or mutant EII HNF-4α-Mut (C) along with shFN or shNC, and then cell culture medium was refreshed with serum-free medium 6 h post-transfection. Luciferase activity was measured 24 h after serum starvation. (D) Huh7 cells were transfected with shFN or shNC. Cells were lysed 48 h post-transfection and HNF-4α levels were determined by qRT-PCR (upper panel) and western blot (lower panel). (E) Huh7 cells were transfected with shFN or shNC along with pHBV and then ChIP analysis was performed to assess HNF-4α binding capability to HBV enhancers 48 h post-transfection. All experiments were repeated at least three times with similar results. Data represent means ± SD, n = 3 (*p<0.05).

### FN Strengthens HBV Enhancer Activity

In addition to the IFN pathway, we also tested whether or not FN regulates liver-specific transcriptional factors that support HBV replication. HBV promoter reporter constructs EI and EII were transfected into Huh7 cells along with pFN. Results from the luciferase activity assays indicated that FN activated both HBV EI and EII (Fig 8A). After screening the effects of FN on the expression of seven transcriptional factors (HNF-4α, peroxisome proliferator-activated receptor α (PPARα), retinoid X receptor α (RXRα), farnesoid X receptor α (FXRα), liver receptor homolog 1 (LRH1), estrogen-related receptor (ERR)β, and ERRγ) which are known to support HBV transcription, we found that only HNF-4α was regulated by FN (S5 Fig). Furthermore, wild-type HBV EI and EII, and HNF-4α-mutant EI and EII constructs (EI HNF-4α-Mut and EII HNF-4α-Mut) were co-transfected into Huh7 cells along with shNC or shFN. Subsequent luciferase activity assays showed that knockdown of FN reduced the activity of both wild-type EI and EI HNF-4α-Mut significantly (Fig 8B). In contrast, knockdown of FN only inhibited the activity of wild-type EII but not EII HNF-4α-Mut (Fig 8C). These data suggest that FN may selectively enhance HNF-4α-mediated HBV EII activity.

We then examined HNF-4α expression by qRT-PCR and western blot analysis before and after the knockdown of FN to verify our previous results. We found that knockdown of FN reduced HNF-4α mRNA and protein levels (Fig 8D). In addition, NC-KD cells and FN-KD cells were transfected with pHBV, and ChIP assays were then performed to test the binding capability of HNF-4α to HBV EI and EII. The results showed that binding of HNF-4α to HBV EII decreased significantly in FN-KD cells compared with NC-KD cells. In contrast, binding of HNF-4α to HBV EI showed scarcely any change (Fig 8E). These results demonstrate that FN promotes HNF-4α-mediated HBV EII, but not EI, activity.

## Discussion

There are few studies regarding the relationship between FN and HBV, but it is suggested that human liver sinusoid FN binds to HBV [16]. In addition, asialoglycoprotein receptor, FN, and HBsAg were found to bind to each other. Both asialoglycoprotein receptor and FN could promote the binding of HBsAg to several cell types [17]. Other studies found that FN accelerates HBV infection in primary cultured fetal hepatocytes in vitro [18], and that protease cleavage or anti-sense oligonucleotide treatment indicate that FN is important for HBV propagation [19, 34]. These previous studies indicate that FN may be involved in HBV expression and replication; however, a detailed mechanism remains unknown. In this study, we found novel roles for FN in facilitating HBV expression and replication. Firstly, HBV upregulated FN expression in both clinical samples and cultured cells. The increased FN then antagonized the endogenous IFN pathway, leading to enhanced viral protein synthesis and HBV DNA replication. Secondly, FN promoted HBV replication and expression by strengthening the recruitment of HNF-4α to HBV EII. Our results reveal the important contributions of host factors to HBV maintenance.

Considering that FN expression is elevated in HBV patients, we used different types of hepatic cell cultures to verify whether the up-regulation of FN was induced by HBV. HBV enhanced FN expression at both mRNA and protein levels *in vitro*, as similar as in HBV patients. Although the induction of FN was only about 2 to 3 folds, it has important physiological significance as FN is one of the most abundant proteins in the hepatocytes and serum[7].

In this study, we constructed a stable FN-KD cell line to investigate the role of FN in HBV replication. HBV expression and replication declined in FN-KD cells, and HBeAg, HBsAg, and HBV DNA were rescued by exogenous FN. Interestingly, the rescued level of HBsAg was significantly higher than that of HBeAg and HBV DNA when compared with their corresponding controls. The HBsAg synthesis pathway is separated from the viral replication pathway, although it is synthesized from the same viral genome as HBV DNA [41]. This may explain why the rescued HBsAg level is not entirely in accordance with HBeAg or HBV DNA.

Hepatitis D virus (HDV) is a small satellite RNA virus of HBV and propagates only when coexisting with HBV. Viral hepatitis D is considered as one of the most severe forms of human viral hepatitis. Although some efforts were made, the mechanisms and therapies for HVD infection remains unclear [42, 43]. HDV markers may reflect the infection of HBV to a certain extent. In previous reports, seven days post HDV infected with Huh7, HepG2, Hepa1–6 and AML-12 cells expressed NTCP, HDAg can be evidently detected [44]. In Human NTCP transgenic mice, HDV RNA reached peak level around 6 day post infection [45]. The time of FN induction was day 9 post HBV infected HepG2-NTCP cells and it is comparable to the reported observation of HDV which may indicate the induction of FN was followed the production of HBV and HDV.

IFN-α remains one of the most effective compounds in HBV therapy even though HBV is considered an immune-resistant virus. Previous study showed that HBV could inhibit the innate responses in HepaRG cells and primary human hepatocytes [46]. In the present study, we identified a previously undescribed role for FN in facilitating HBV expression and replication by hampering the production of IFN-α and ISGs. The liver is not only an immune organ but also plays a role in immune tolerance [47], and we found that FN inhibits IFN-α and ISG production. In addition, the adaptor proteins in the IFN pathway, the TAK1-TAB complex, were found to interact with FN, and TAK1 phosphorylation was inhibited by FN. According to the results in Fig 5D, FN did not significantly influence IFN-α promoter activity without virus infection. Similarly, as shown in Fig 6B, the interaction between FN and the TAK-TAB complex was much weaker before virus incubation, suggesting that HBV promotes the expression of FN and triggers the interaction between FN and the TAK1-TAB complex to maintain its expression and replication.

We found that the C-terminal domain of the FN functional domain had the strongest interaction with TAB3 in the TAK1-TAB complex. The other segments also had some interaction with the TAK1-TAB complex, possibly due to the peculiar structure of FN. Cellular FN has many repeating domains with many similarities and only few significant differences among the domains [35, 48, 49], and it has also been suggested that more than one domain of FN binds to heparin and fibrin [50].

As a general rule, host cells can sense the viral infection and activate innate/adaptive immue response. However, some virus including HBV do not trigger a strong immune response nor induce an obvious production of type I IFN [51]. Previous studies have showed that HBV inhibits antiviral immunity, induces immune tolerance as well as inhibits type I IFN production and signaling [52]. Besides, HBV protein components can impair type I IFN transcription in different ways [53–55].Because of these reasons, we used Sendai virus as an alternative agonist to stimulate IFN-α production to study the mechanism of FN in interfering IFN signaling.

In addition to inhibition of IFN-α production, HBV EI and EII were also activated by FN to facilitate HBV expression and replication. Interestingly, FN selectively influenced the HNF-4α binding capacity to HBV EII but not HBV EI. This result may be partly attributed to the fact that there is only one transcriptional factor (HNF-4α) binding site in the 1660–1680 bp region in HBV EII [56], but there are a few transcription factor (HNF-4α, PPARα, and RXRα) binding sites in the HBV EI 990–1020 bp region [57]. These other transcriptional factors may have an alternative role, which necessitates further investigation.

**Fig 9:**
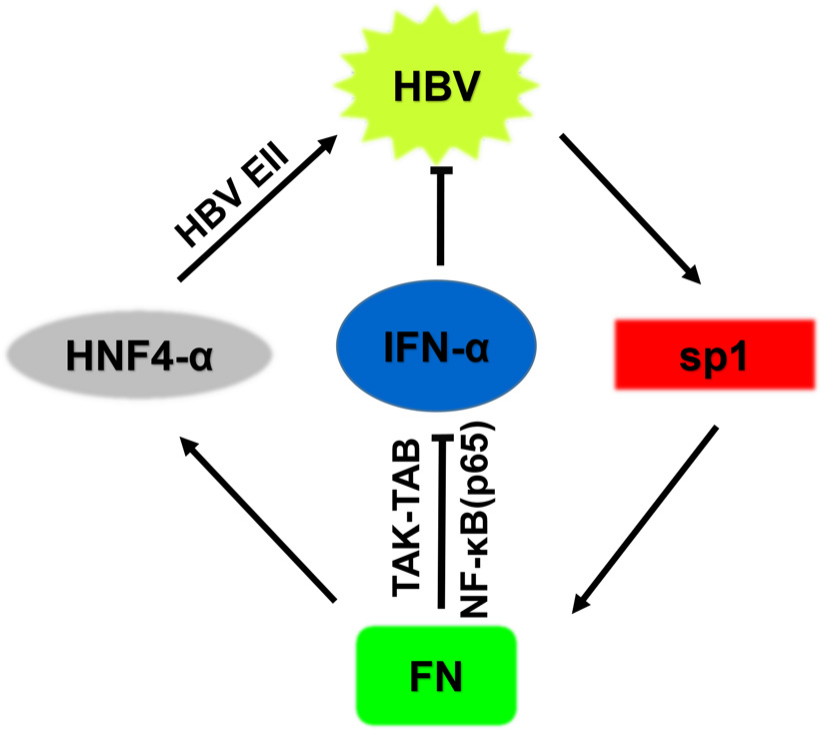
A schematic figure shows the relationship between FN and HBV. HBV increases FN expression through activation of Sp1. In turn, induced FN facilitates HBV replication and expression via two distinct mechanisms: 1) FN binds to the TAK-TAB complex, inhibits phosphorylation of TAK1 and reduces the nuclear translocation of NF-κB p65 to decrease endogenous IFN-α production; 2) FN activates HBV EII activity by mediating the binding of liver-specific factor HNF-4α.

In the present study, we uncovered the mechanisms by which FN facilitates HBV replication. As shown in Fig 9, HBV increased FN expression through activation of Sp1. In turn, the induced FN facilitates HBV expression and replication through two distinct mechanisms. On the one hand, FN binds to the TAK1-TAB complex, inhibits TAK1 phosphorylation and reduces the nuclear translocation of NF-κB p65 to decrease IFN-α production; on the other hand, FN increases HNF-4α-mediated HBV EII activity. Together with those of previous studies, our results suggest that FN may be a potential and attractive target for HBV therapy.

## Supporting Information

S1 Fig MTT assay shows that over-expression or knockdown of FN do not significantly change the cell viability. (PDF)

S2 Fig FN enhance the intracellular HBsAg expression level. (PDF)

S3 Fig HBL enhances the interaction between FN and TAB3. (PDF)

S4 Fig Intracellular FN interacts with TAK1. (PDF)

S5 Fig Screening of FN regulated nuclear factors that support HBV replication. (PDF)

S6 Fig Original blots in Fig 1. (PDF)

S7 Fig Original blots in Fig 2. (PDF)

S8 Fig Original blots in Fig 4. (PDF)

S9 Fig Original blots in Fig 5. (PDF)

S10 Fig Original blots in Fig 6. (PDF)

S11 Fig Original blots in Fig 7. (PDF)

S12 Fig Original blots in Fig 8. (PDF)

S1 Table Baseline Characteristics of HBV-Infected Patients and Healthy individuals in Fig 1A and 1B. (PDF)

S2 Table Baseline Characteristics of HBV-Infected Patients and Healthy individuals in Fig 1C. (PDF)

S3 Table Sequence of shRNA, Q-PCR or RT-PCR primers. (PDF)
