# Supplementary material for: Hepatitis B Virus Stimulated Fibronectin Facilitates Viral Maintenance and Replication through Two Distinct Mechanisms
Source: PLoS One. 2016 Mar 29;11(3):e0152721. doi: 10.1371/journal.pone.0152721 (PMC4811540; doi:10.1371/journal.pone.0152721)
Supplement: S1 Fig — (PDF) [file pone.0152721.s001.pdf]

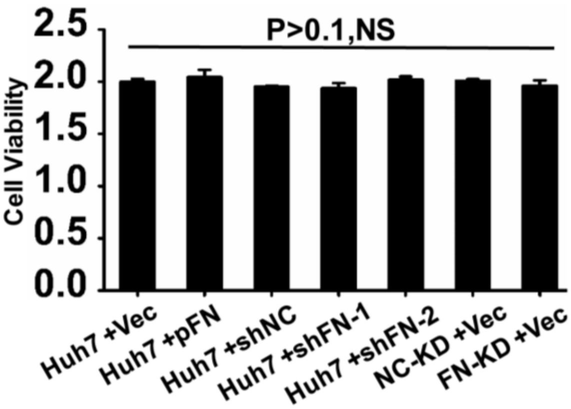

S1 Fig. MTT assay shows that over-expression or knockdown of FN do not significantly change the cell viability. (A) MTT assay analysis of cells transfected with pFN, shFN or transduced with shFN-lentivirus. All experiments were repeated at least three times with consistent results. Bar graphs represent the means ± SD, n = 3 (*$P < 0.05$).
