# Supplementary material for: Hepatitis B Virus Stimulated Fibronectin Facilitates Viral Maintenance and Replication through Two Distinct Mechanisms
Source: PLoS One. 2016 Mar 29;11(3):e0152721. doi: 10.1371/journal.pone.0152721 (PMC4811540; doi:10.1371/journal.pone.0152721)
Supplement: S2 Fig — (PDF) [file pone.0152721.s002.pdf]

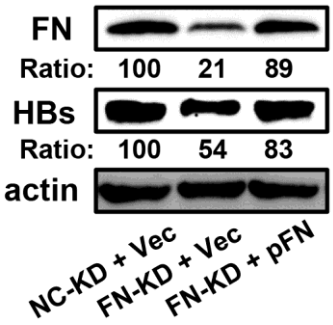

S2 Fig. FN enhance the intracellular HBsAg expression level.

NC-KD or FN-KD cells were transfected with empty vector or pFN along with pHBV. At 48 h after transfection, the intracellular HBsAg protein level was determined by Western Blot. Numbers below the blots are the quantified optical density; control blots were set as 100. All experiments were repeated at least three times with similar results.
