# Supplementary material for: Hepatitis B Virus Stimulated Fibronectin Facilitates Viral Maintenance and Replication through Two Distinct Mechanisms
Source: PLoS One. 2016 Mar 29;11(3):e0152721. doi: 10.1371/journal.pone.0152721 (PMC4811540; doi:10.1371/journal.pone.0152721)
Supplement: S3 Fig — (PDF) [file pone.0152721.s003.pdf]

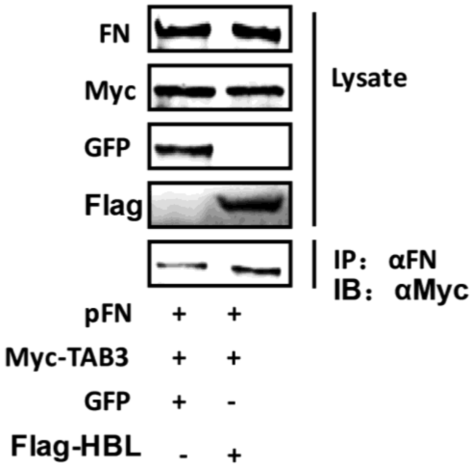


**S3 Fig. HBL enhances the interaction between FN and TAB3.** HEK293 cells were transfected with constructs expressing GFP or Flag-HBL along with pFN and Myc-TAB3. Cells were lysed 24 h post-transfection and the lysates were immunoprecipitated with anti-FN. The immunoprecipitates were analyzed by immunoblot with anti-Myc. All experiments were repeated at least three times with similar results.
