# Supplementary material for: Hepatitis B Virus Stimulated Fibronectin Facilitates Viral Maintenance and Replication through Two Distinct Mechanisms
Source: PLoS One. 2016 Mar 29;11(3):e0152721. doi: 10.1371/journal.pone.0152721 (PMC4811540; doi:10.1371/journal.pone.0152721)
Supplement: S4 Fig — (PDF) [file pone.0152721.s004.pdf]

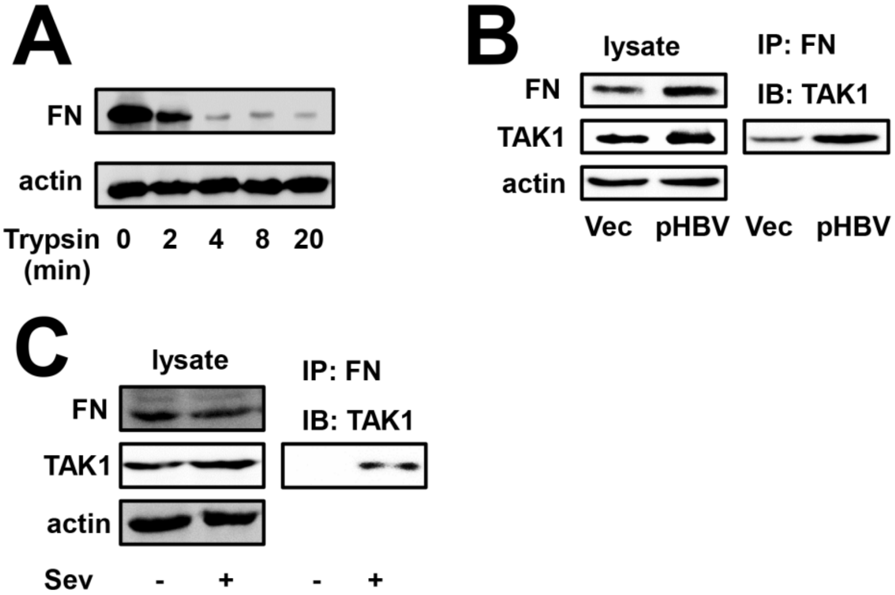

S4 Fig. Intracellular FN interacts with TAK1. (A) Huh7 cells were digested with trypsin (PBS containing 0.2% trypsin and 0.02% EDTA) for indicated time and then neutralized with 10% FBS DMEM. Cells lysates were analyzed FN expression by western blot. (B) Huh7 cells were transfected with empty vector or pHBV. Cells were lysed 48 h post-transfection and the lysates were immunoprecipitated with anti-FN. The immunoprecipitates were analyzed by immunoblot with anti-TAK1. (C) Suspension cell THP-1 were mock infected or SeV (MOI=1) infected, cells were lysed 24 h post infection and the lysates were immunoprecipitated with anti-FN. The immunoprecipitates were analyzed by immunoblot with anti-TAK1. All experiments were repeated at least three times with similar results.
