# Supplementary material for: Hepatitis B Virus Stimulated Fibronectin Facilitates Viral Maintenance and Replication through Two Distinct Mechanisms
Source: PLoS One. 2016 Mar 29;11(3):e0152721. doi: 10.1371/journal.pone.0152721 (PMC4811540; doi:10.1371/journal.pone.0152721)
Supplement: S5 Fig — (PDF) [file pone.0152721.s005.pdf]

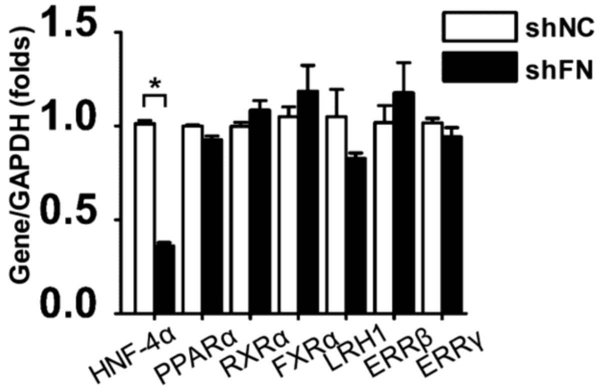


**S5 Fig. Screening of FN regulated nuclear factors that support HBV replication.**

Huh7 cells were transfected with shFN or shNC. Cells were lysed 48 h post-transfection and the expression of indicated nuclear factors were determined by qRT-PCR. Bar graphs represent the means ± SD, n = 3 (*$P$ < 0.05).
