# Supplementary figures and images for: Hepatitis B Virus Stimulated Fibronectin Facilitates Viral Maintenance and Replication through Two Distinct Mechanisms
Source: PLoS One. 2016 Mar 29;11(3):e0152721. doi: 10.1371/journal.pone.0152721 (PMC4811540; doi:10.1371/journal.pone.0152721)

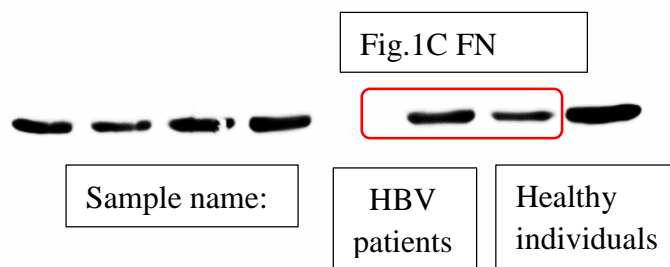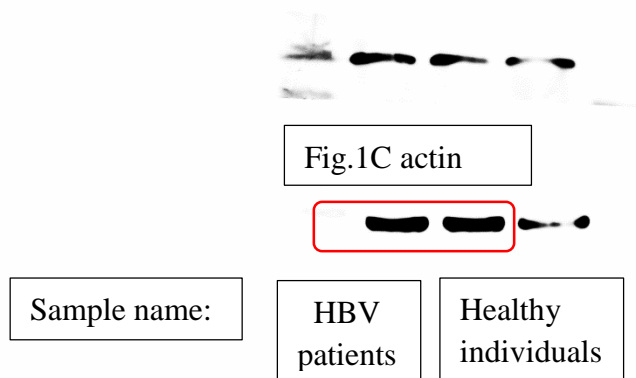

**S6 Fig. Original blots in Fig 1.**

Supplement: S6 Fig — (PDF) [file pone.0152721.s006.pdf]

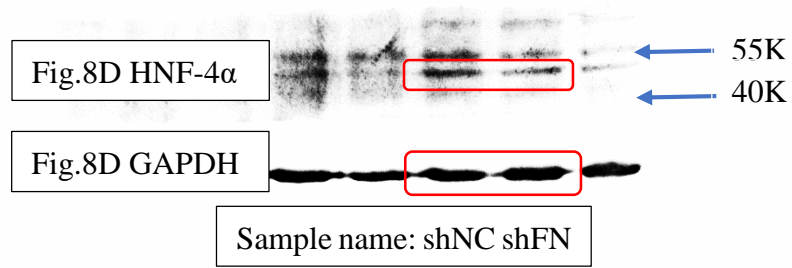

**S12 Fig. Original blots in Fig 8.**

Supplement: S12 Fig — (PDF) [file pone.0152721.s012.pdf]
