# Supplementary material for: Hepatitis B Virus Stimulated Fibronectin Facilitates Viral Maintenance and Replication through Two Distinct Mechanisms
Source: PLoS One. 2016 Mar 29;11(3):e0152721. doi: 10.1371/journal.pone.0152721 (PMC4811540; doi:10.1371/journal.pone.0152721)
Supplement: S7 Fig — (PDF) [file pone.0152721.s007.pdf]

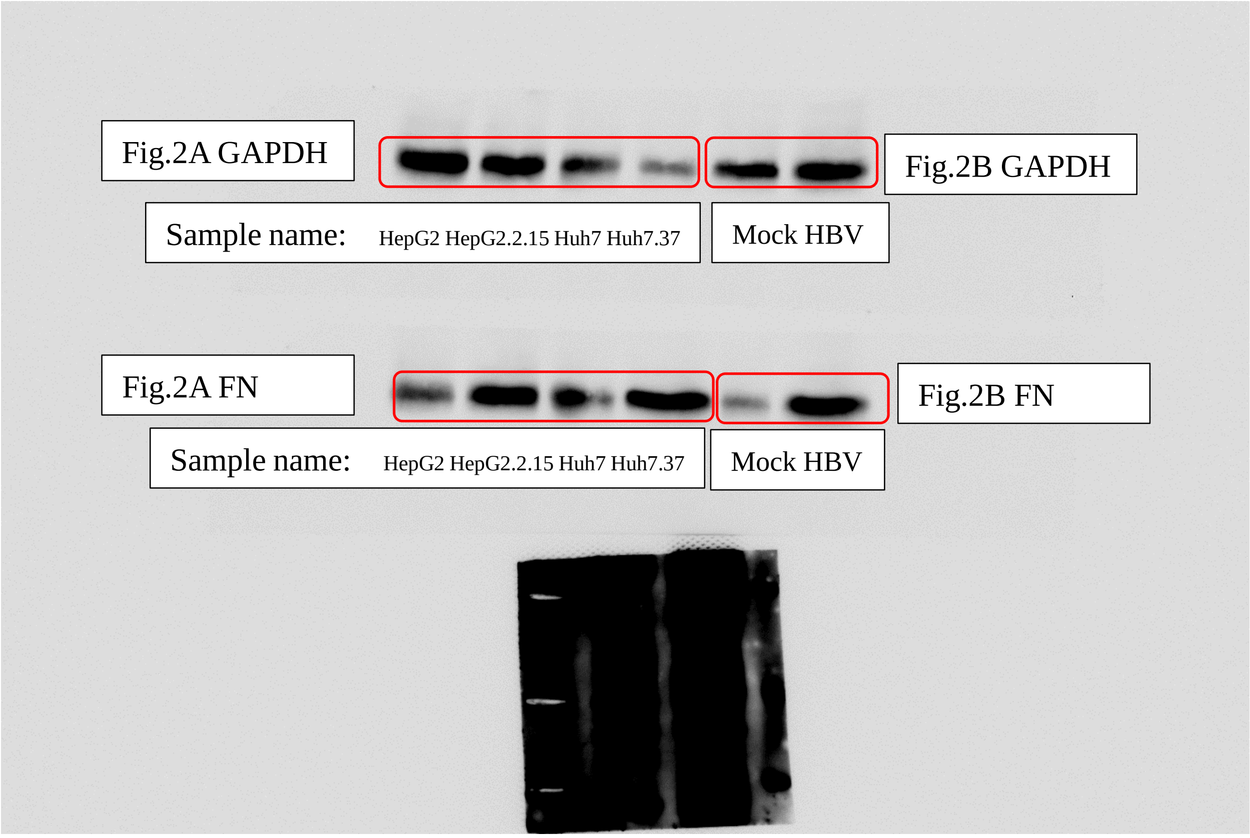

Fig.2A GAPDH
Fig.2B GAPDH
Sample name: HepG2 HepG2.2.15 Huh7 Huh7.37
Mock HBV
Fig.2A FN
Fig.2B FN
Sample name: HepG2 HepG2.2.15 Huh7 Huh7.37
Mock HBV

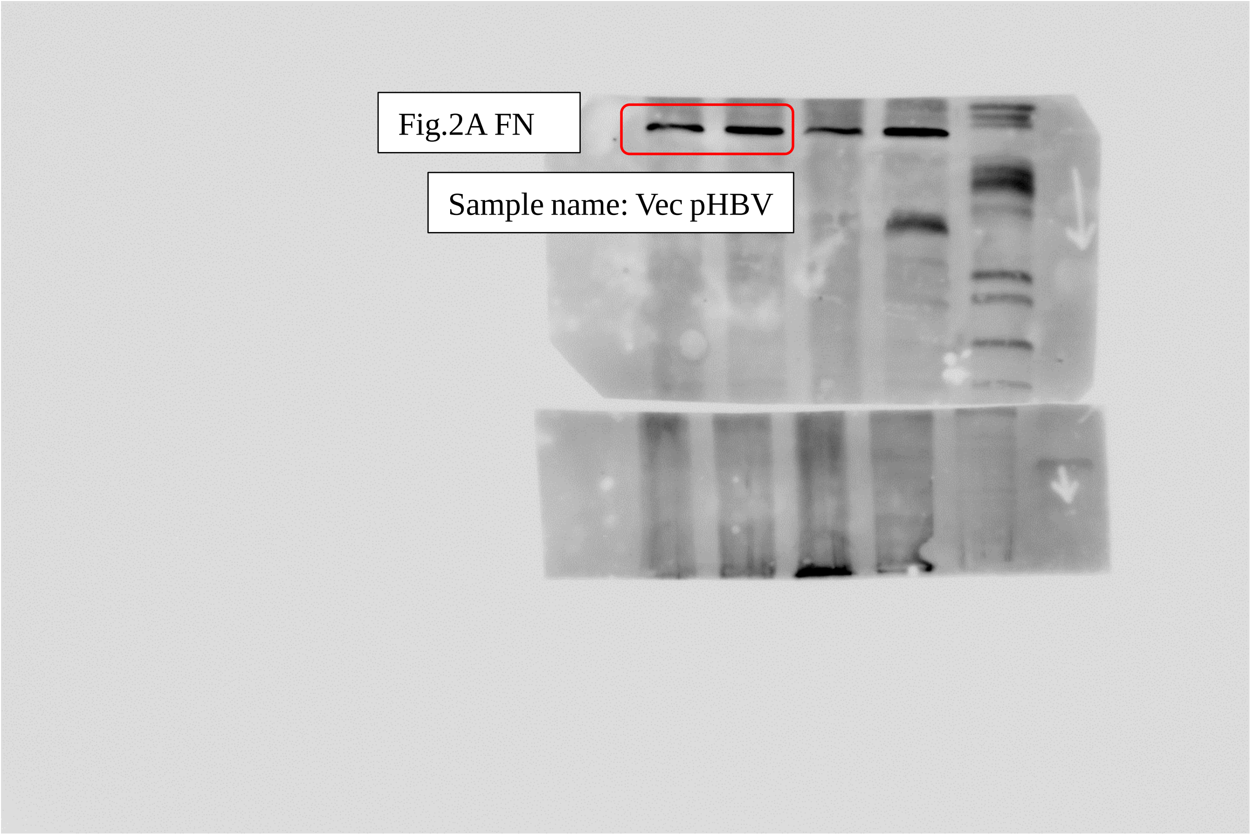
Fig.2A FN
Sample name: Vec pHBV

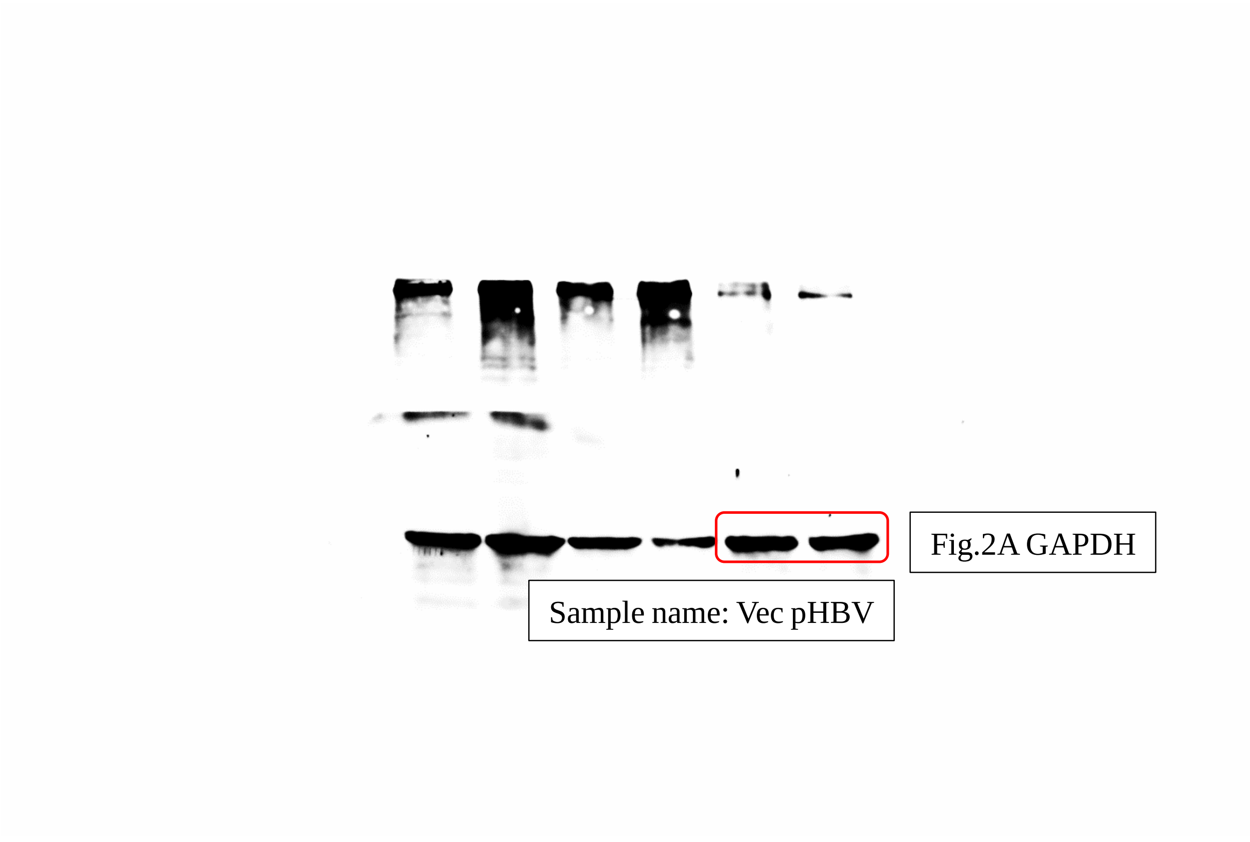
Fig.2A GAPDH
Sample name: Vec pHBV

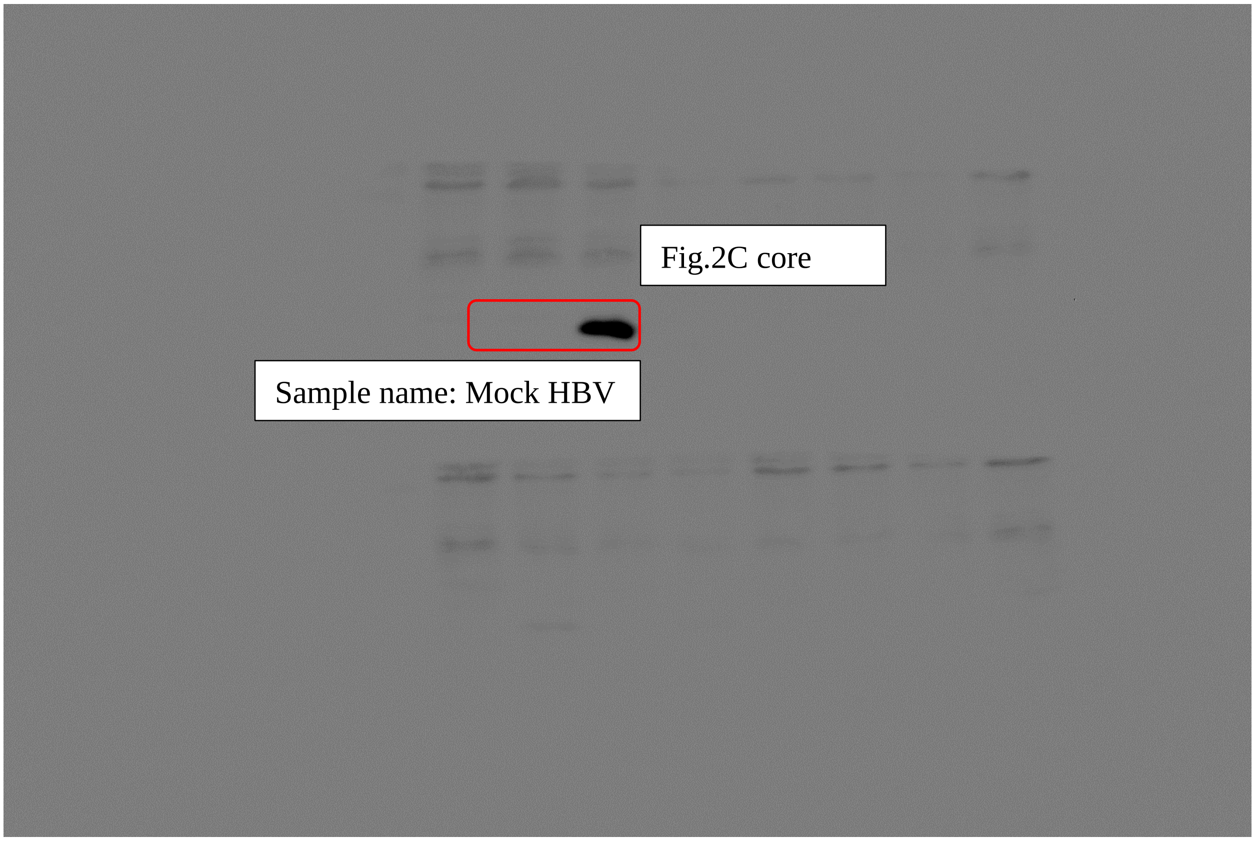
Fig.2C core
Sample name: Mock HBV

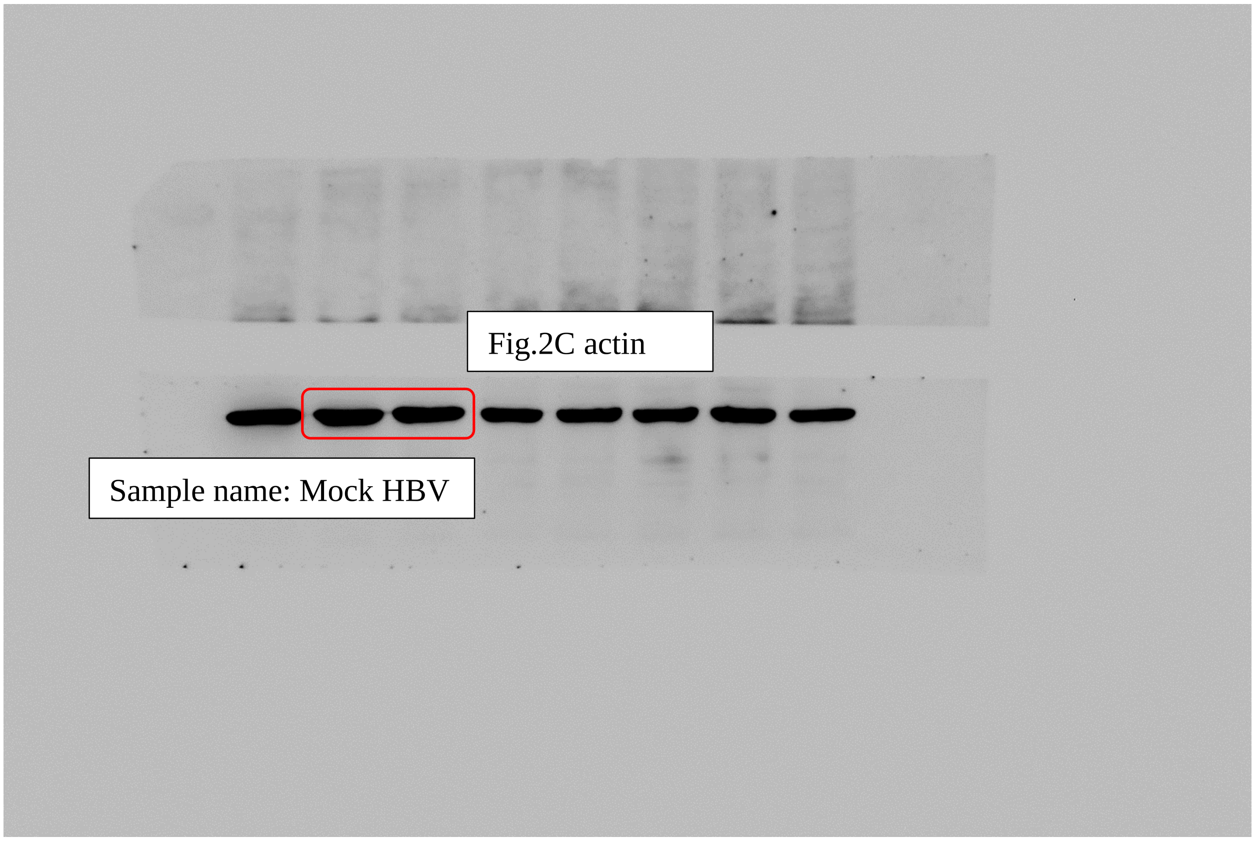
Fig.2C actin
Sample name: Mock HBV

S7 Fig. Original blots in Fig 2.
