# Supplementary material for: Hepatitis B Virus Stimulated Fibronectin Facilitates Viral Maintenance and Replication through Two Distinct Mechanisms
Source: PLoS One. 2016 Mar 29;11(3):e0152721. doi: 10.1371/journal.pone.0152721 (PMC4811540; doi:10.1371/journal.pone.0152721)
Supplement: S8 Fig — (PDF) [file pone.0152721.s008.pdf]

Fig.4D FN

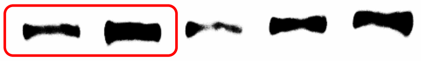

Sample name: Vec pFN

Fig.4D GAPDH

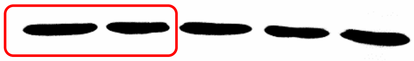

Sample name: Vec pFN

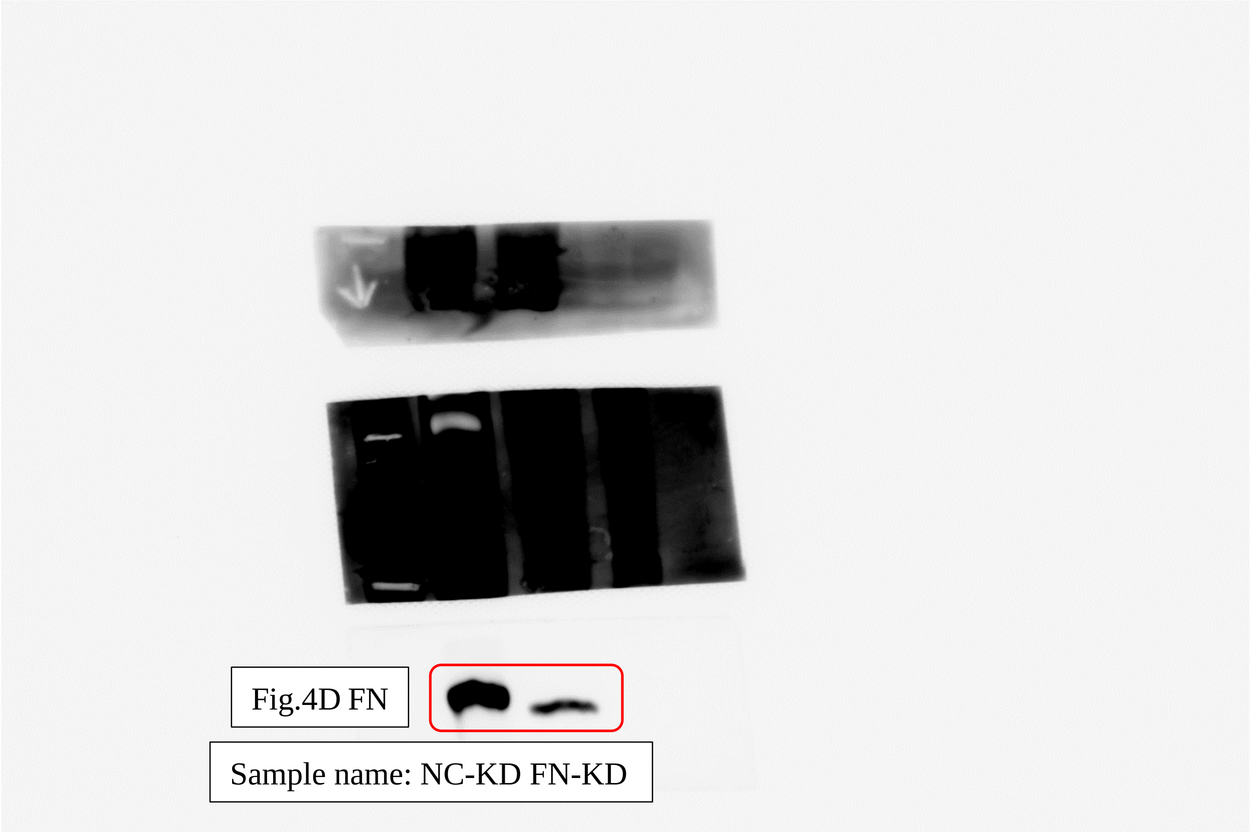
Fig.4D FN
Sample name: NC-KD FN-KD

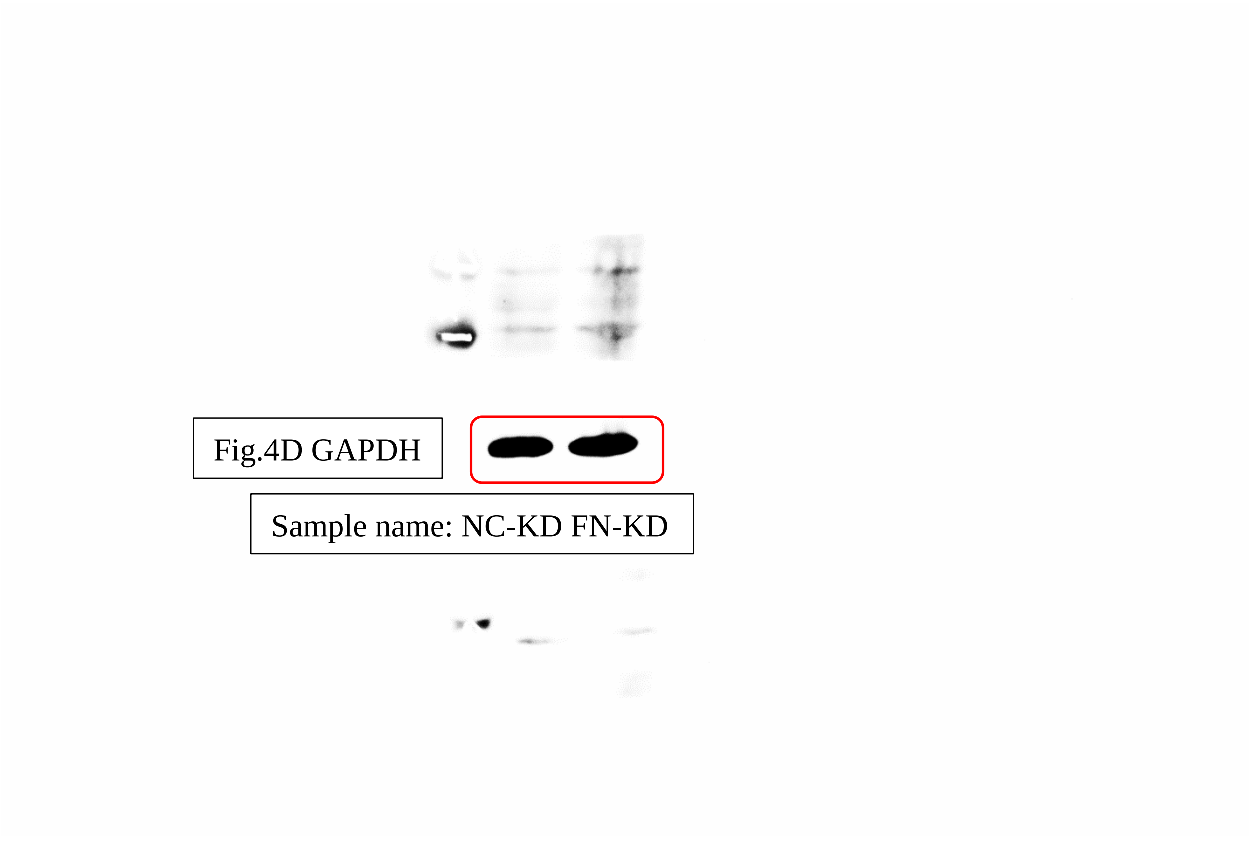
Fig.4D GAPDH
Sample name: NC-KD FN-KD

Fig.4D FN

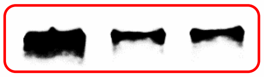

Sample name: shNC shFN-1 shFN-2

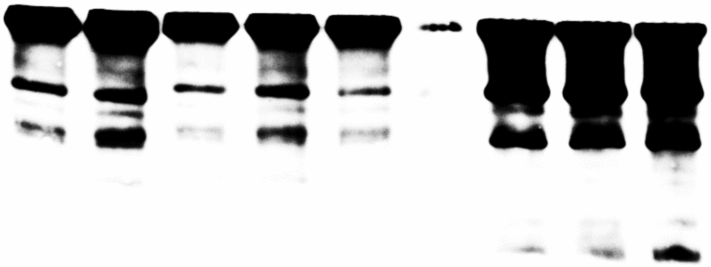

Fig.4D GAPDH

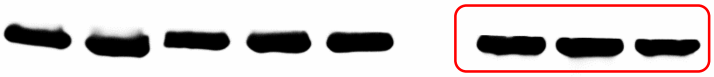

Sample name: shNC shFN-1 shFN-2

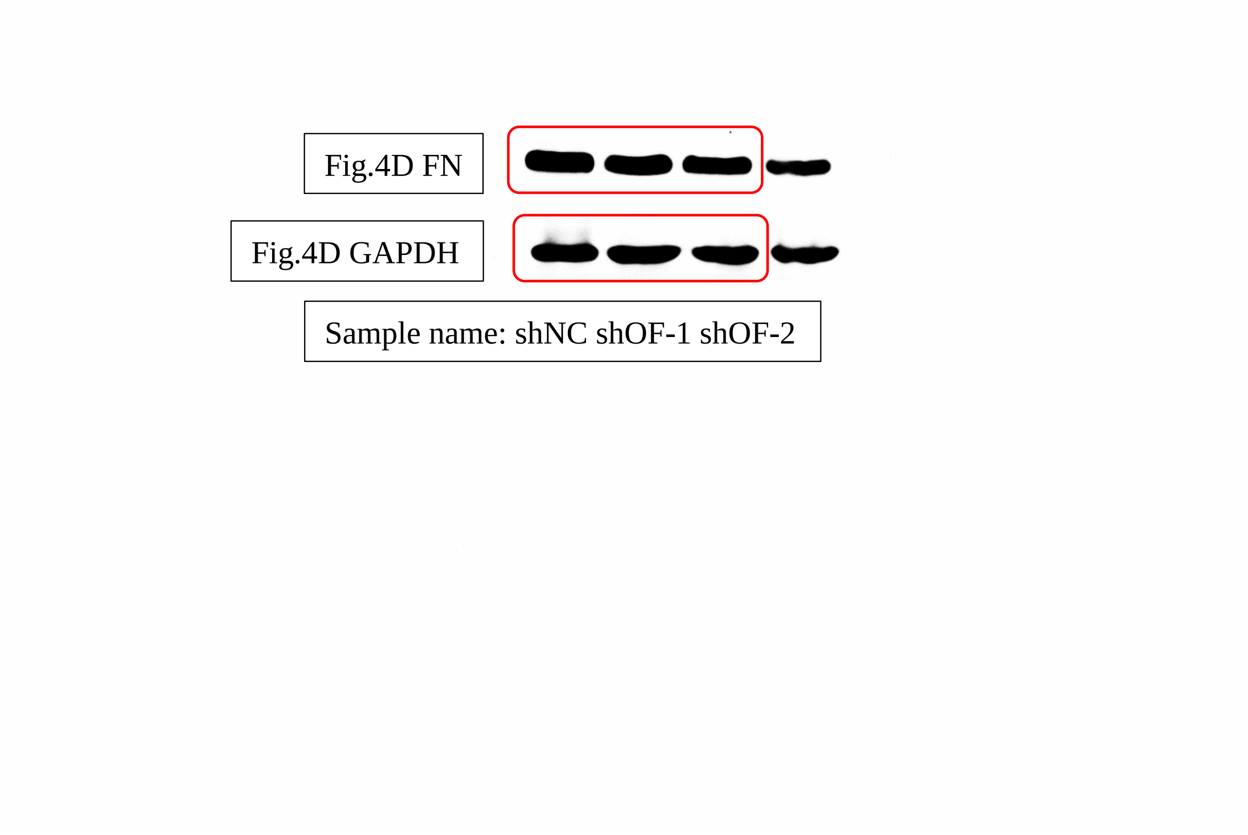

S8 Fig. Original blots in Fig 4.
