# Supplementary material for: Hepatitis B Virus Stimulated Fibronectin Facilitates Viral Maintenance and Replication through Two Distinct Mechanisms
Source: PLoS One. 2016 Mar 29;11(3):e0152721. doi: 10.1371/journal.pone.0152721 (PMC4811540; doi:10.1371/journal.pone.0152721)
Supplement: S9 Fig — (PDF) [file pone.0152721.s009.pdf]

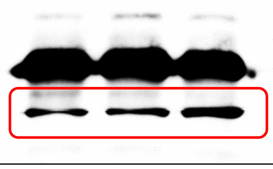
Fig.5B OAS2
Sample name: Sev - + +
NC-KD NC-KD FN-KD

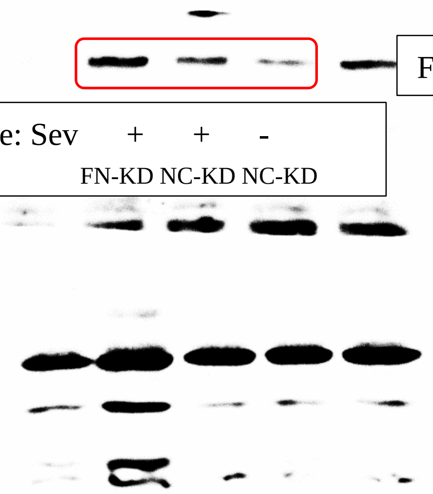
Fig.5B MxA
Sample name: Sev + + -
FN-KD NC-KD NC-KD

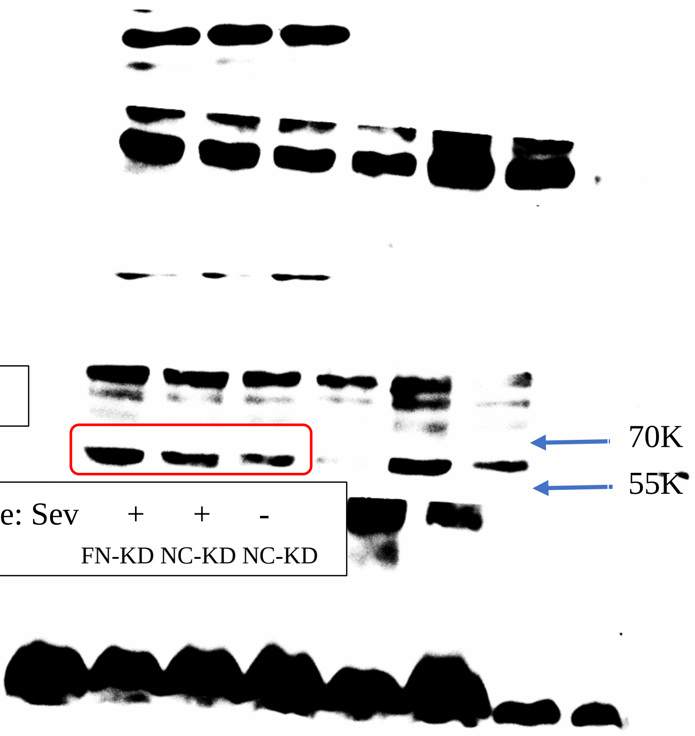

Fig.5B PKR

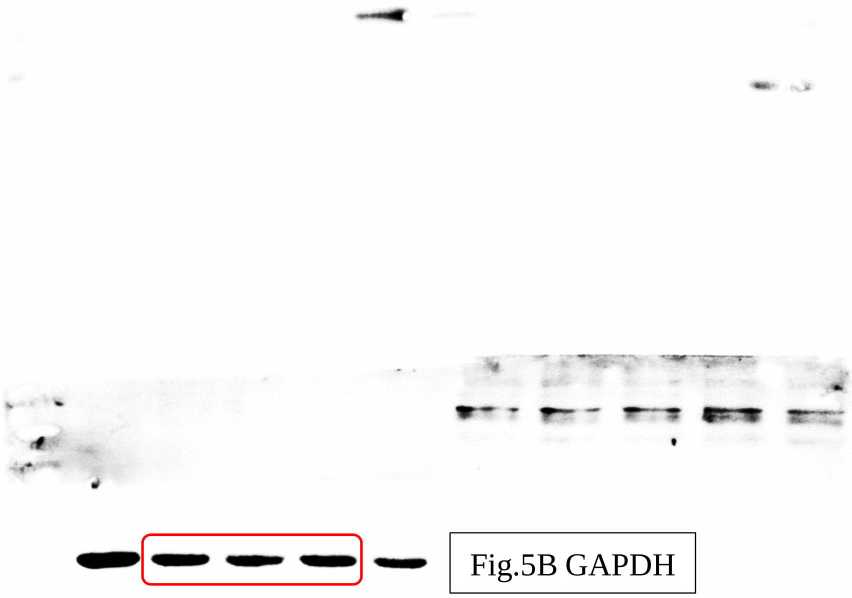

Fig.5B GAPDH

S9 Fig. Original blots in Fig 5.
