# Supplementary material for: Hepatitis B Virus Stimulated Fibronectin Facilitates Viral Maintenance and Replication through Two Distinct Mechanisms
Source: PLoS One. 2016 Mar 29;11(3):e0152721. doi: 10.1371/journal.pone.0152721 (PMC4811540; doi:10.1371/journal.pone.0152721)
Supplement: S10 Fig — (PDF) [file pone.0152721.s010.pdf]

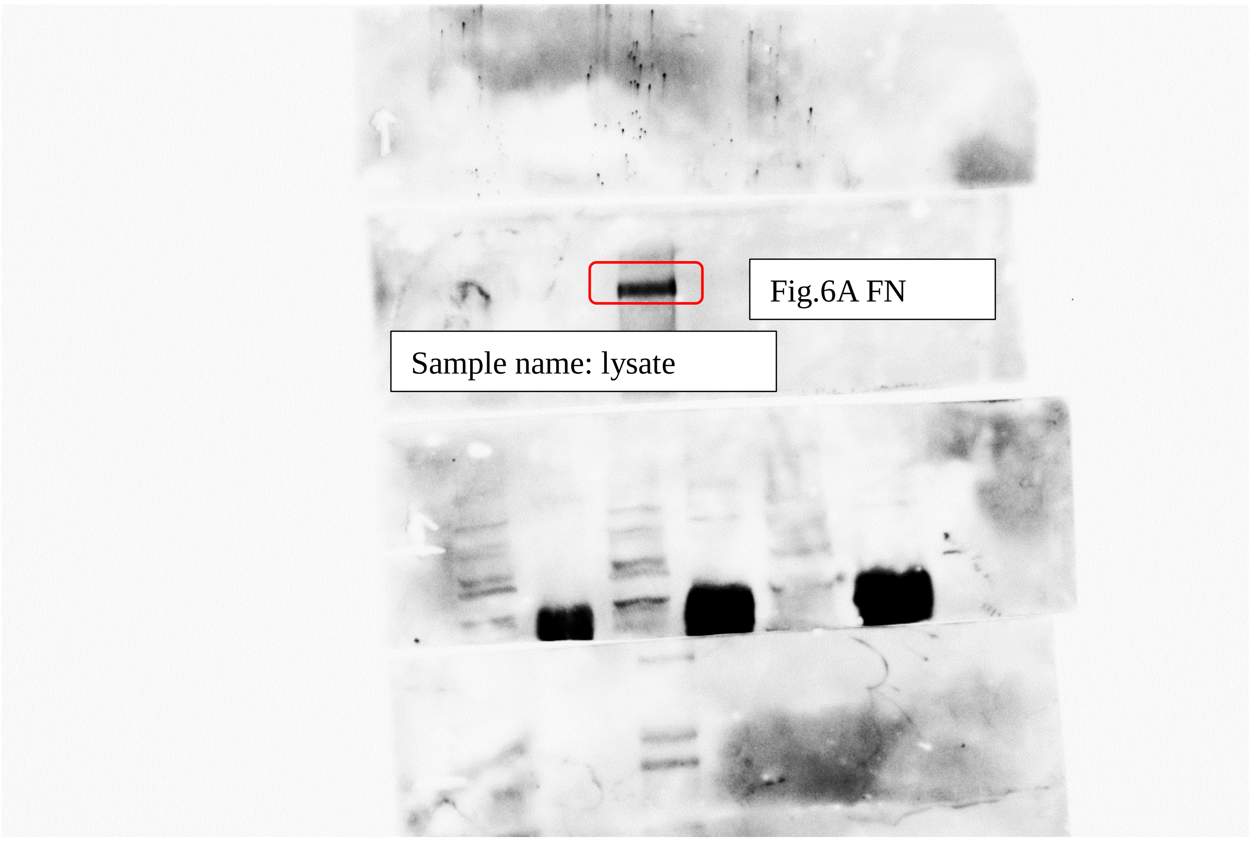
Fig.6A FN
Sample name: lysate

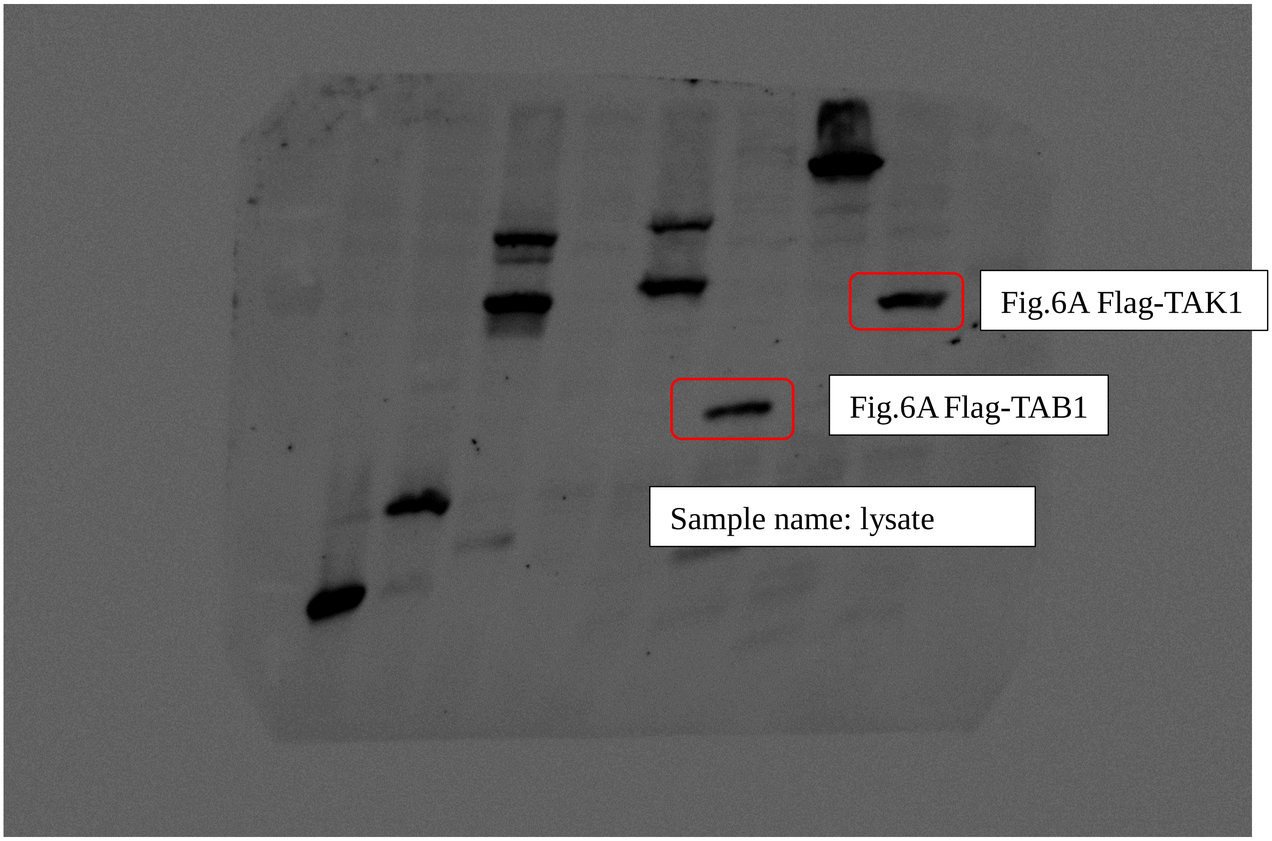
Fig.6A Flag-TAK1
Fig.6A Flag-TAB1
Sample name: lysate

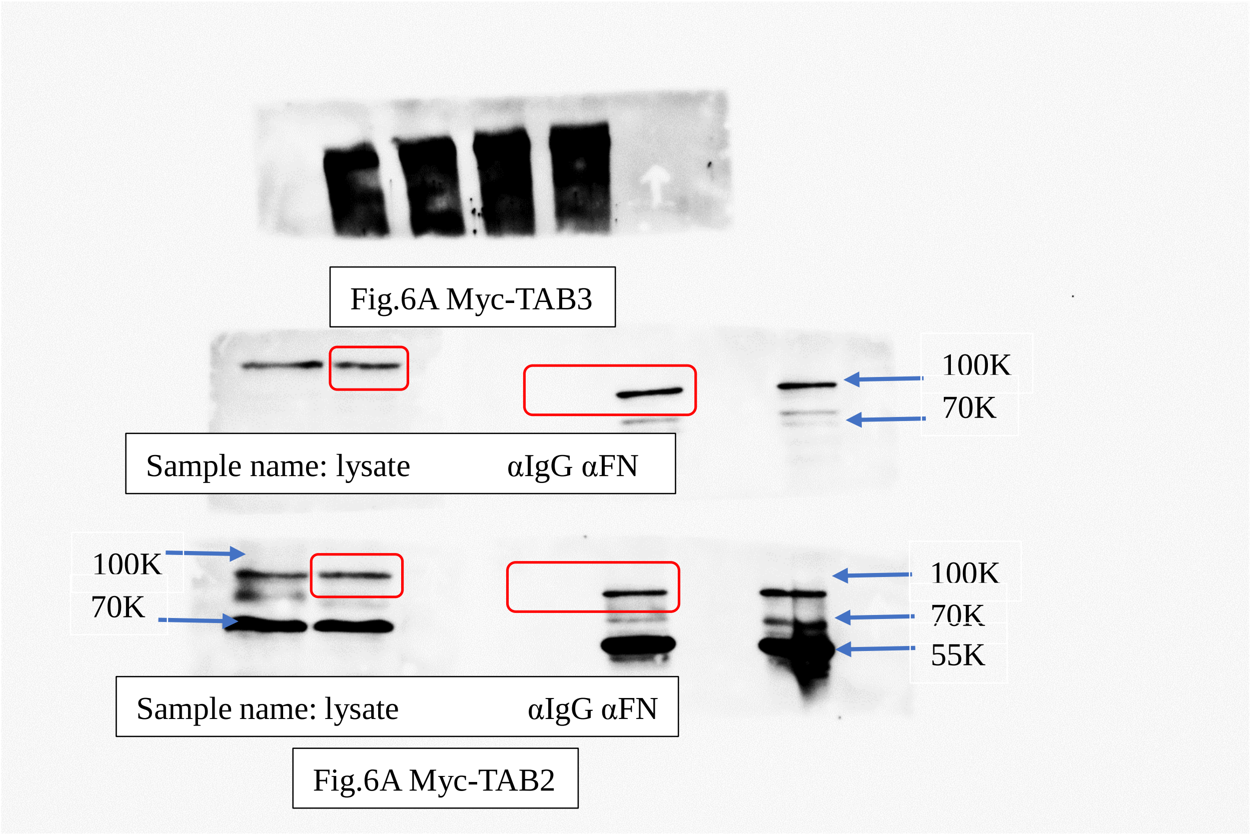
Fig.6A Myc-TAB3
100K
70K
Sample name: lysate αIgG αFN
100K
70K
100K
70K
55K
Sample name: lysate αIgG αFN
Fig.6A Myc-TAB2

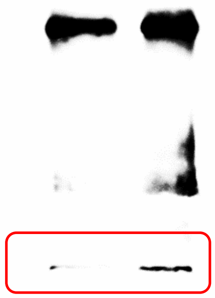

Fig.6A Flag-TAK1

Sample name: αIgG αFN

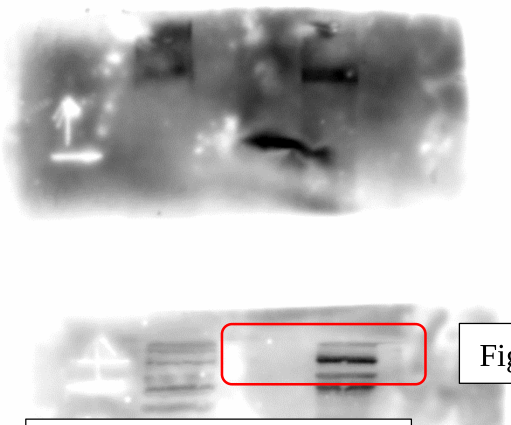

Fig.6A Flag-TAB1

Sample name: αIgG αFN

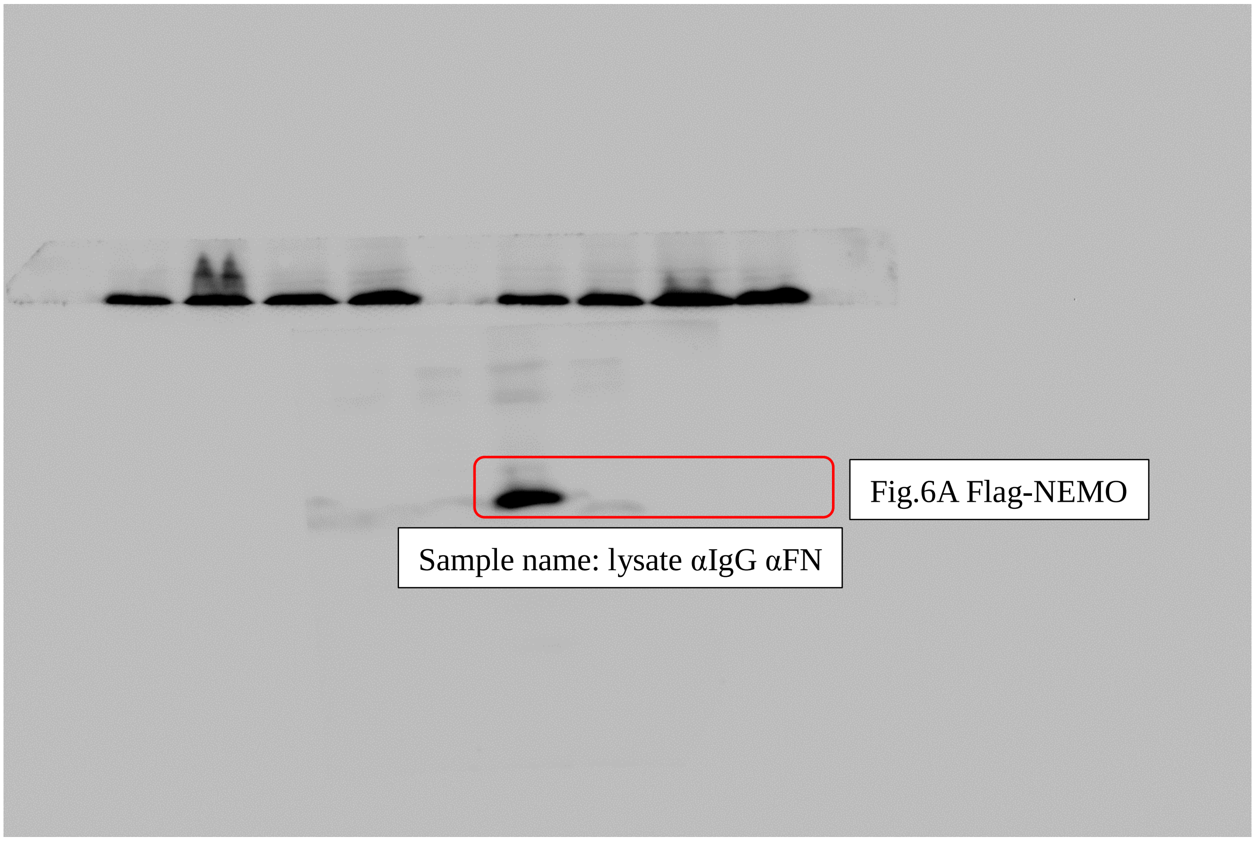
Fig.6A Flag-NEMO
Sample name: lysate αIgG αFN

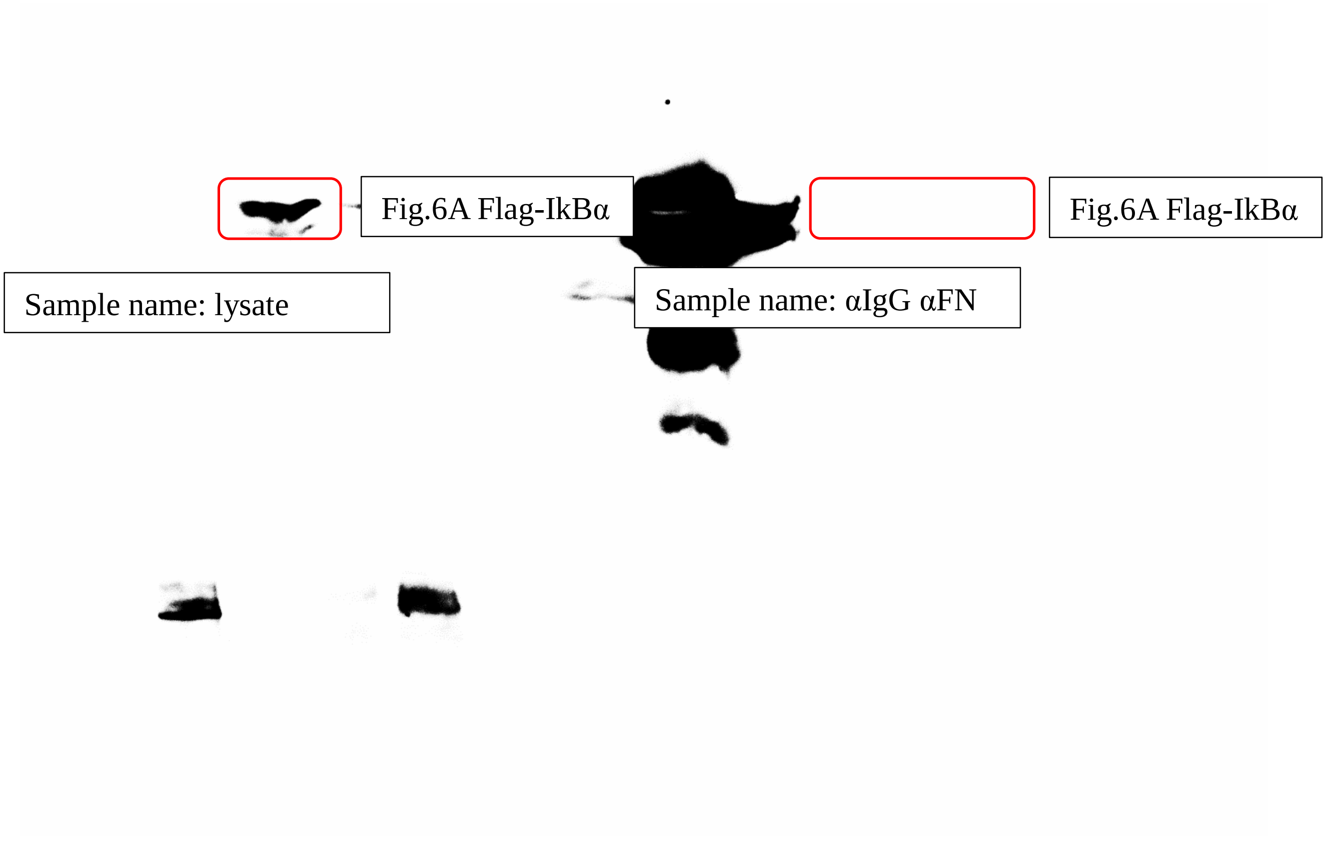
Fig.6A Flag-IkBα
Fig.6A Flag-IkBα
Sample name: lysate
Sample name: αIgG αFN

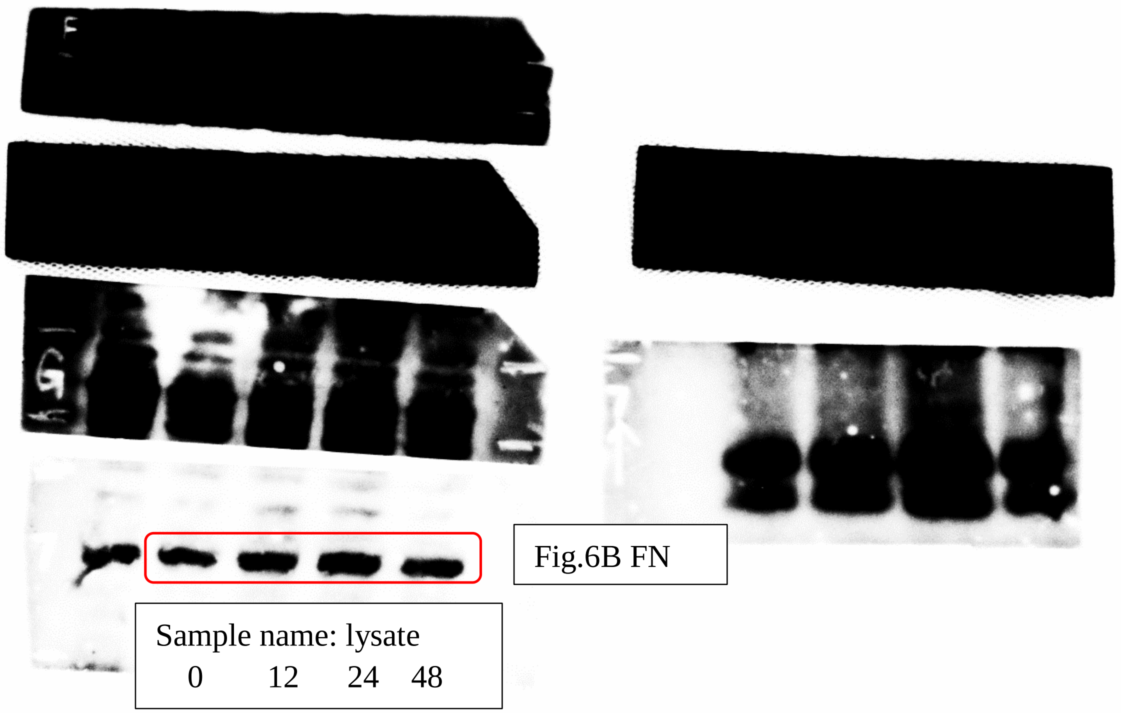

Fig.6B FN

Sample name: lysate
0 12 24 48

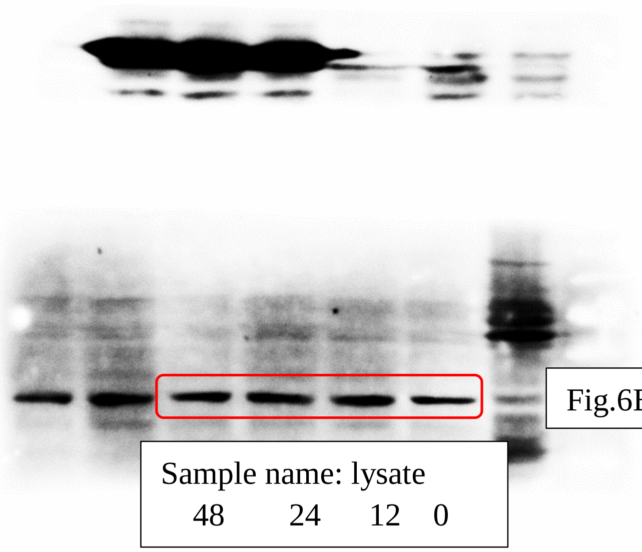
Fig.6B TAK1
Sample name: lysate
48 24 12 0

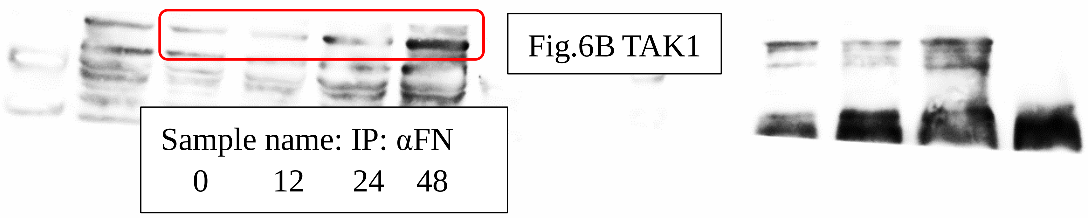
Fig.6B TAK1
Sample name: IP: αFN
0 12 24 48

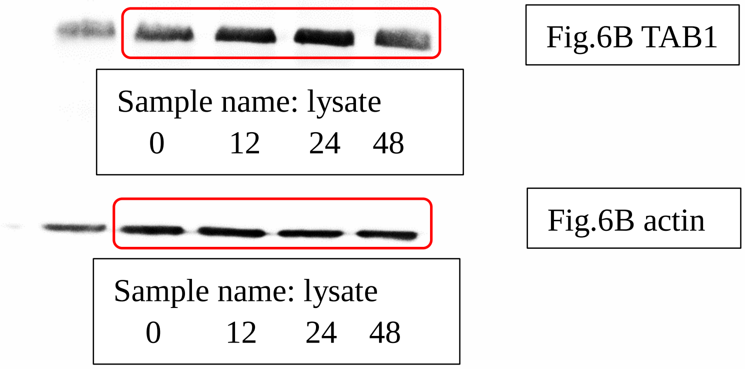
Fig.6B TAB1
Sample name: lysate
0 12 24 48
Fig.6B actin
Sample name: lysate
0 12 24 48

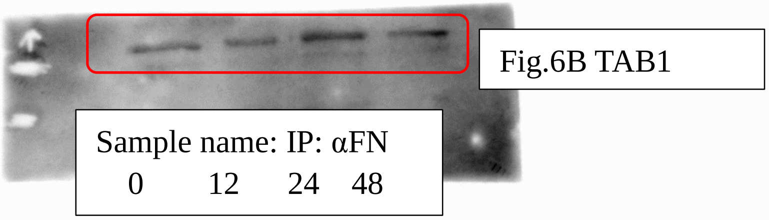
Fig.6B TAB1
Sample name: IP: αFN
0 12 24 48

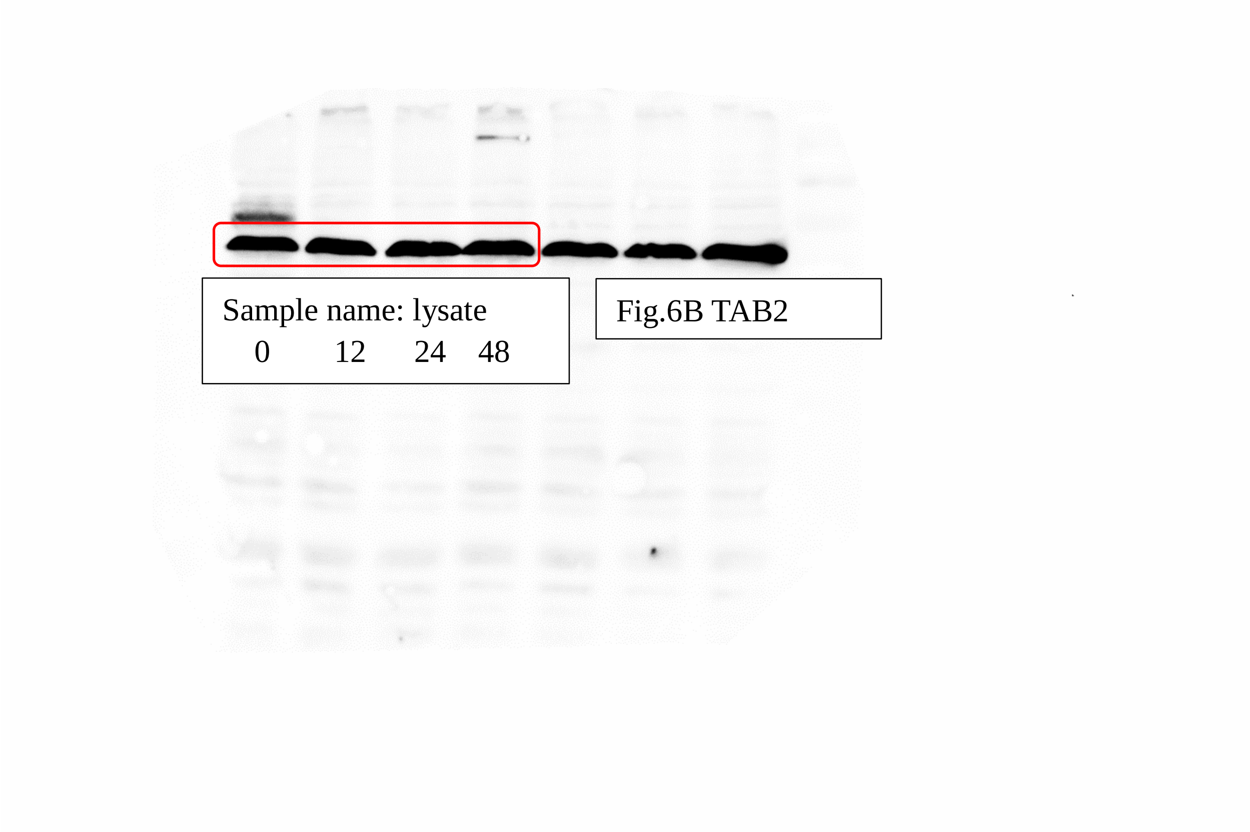
Sample name: lysate
0 12 24 48
Fig.6B TAB2

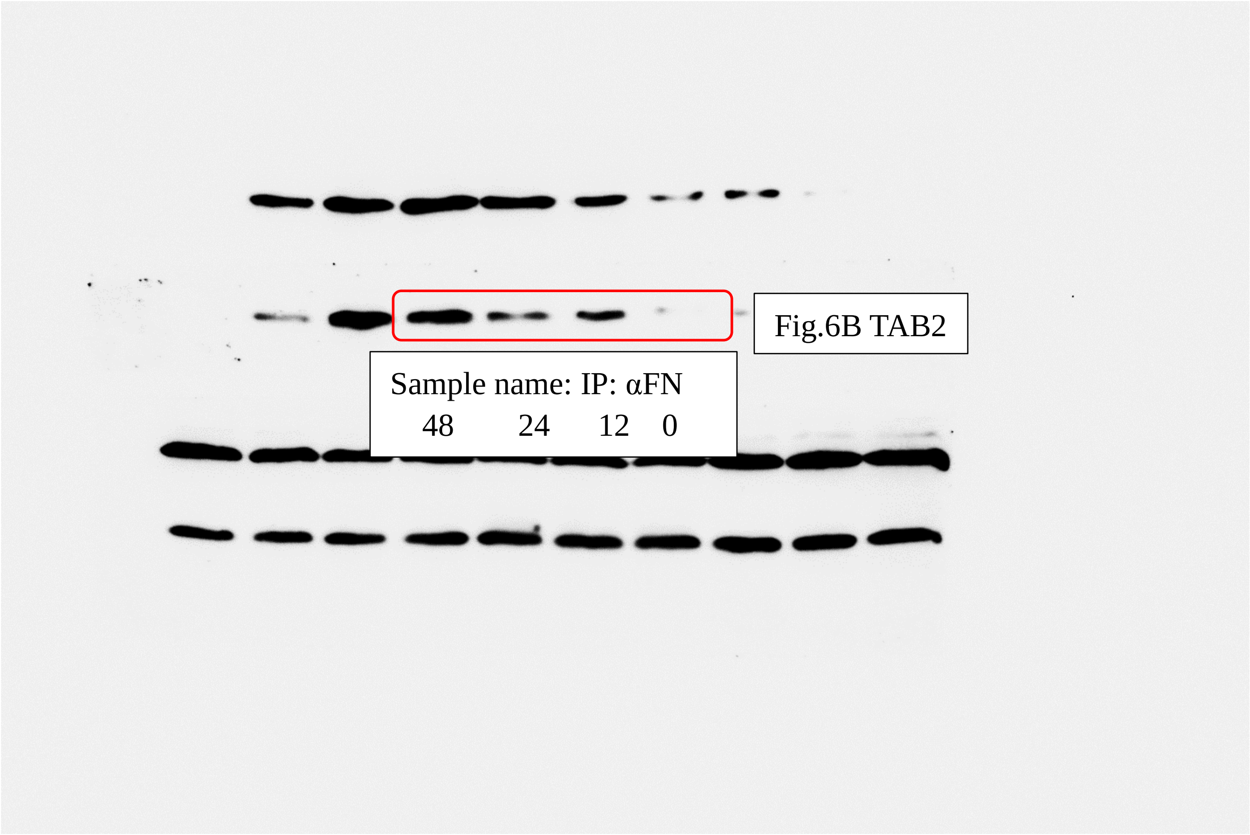
Fig.6B TAB2
Sample name: IP: αFN
48 24 12 0

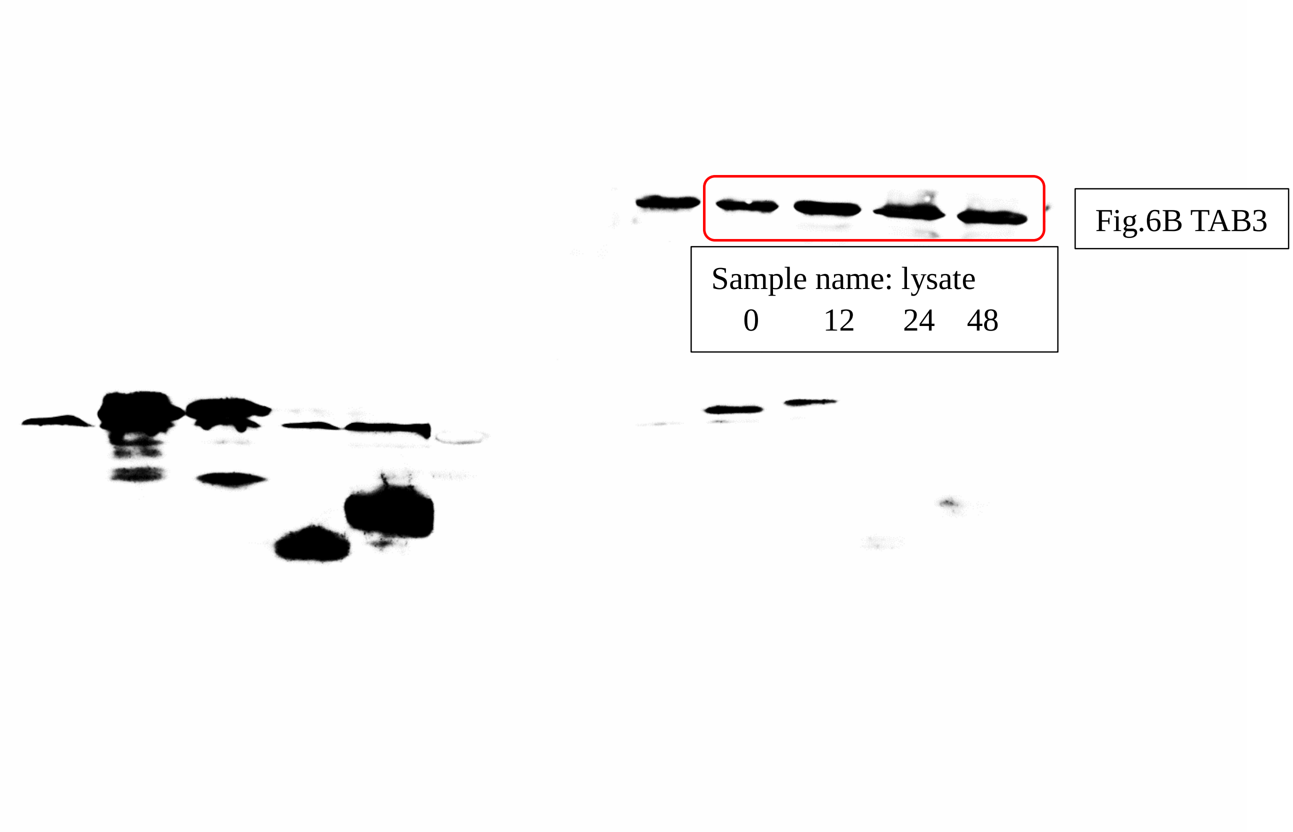
Fig.6B TAB3
Sample name: lysate
0 12 24 48

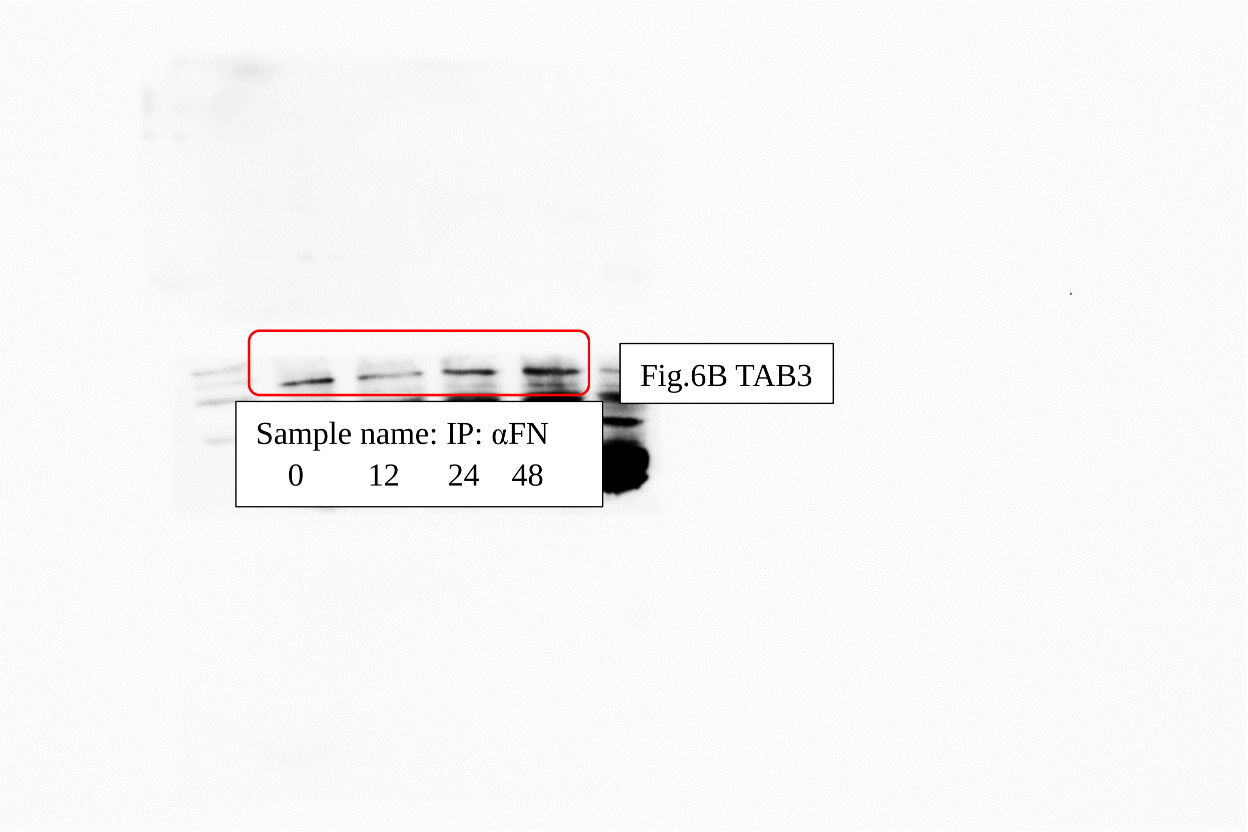
Fig.6B TAB3
Sample name: IP: αFN
0 12 24 48

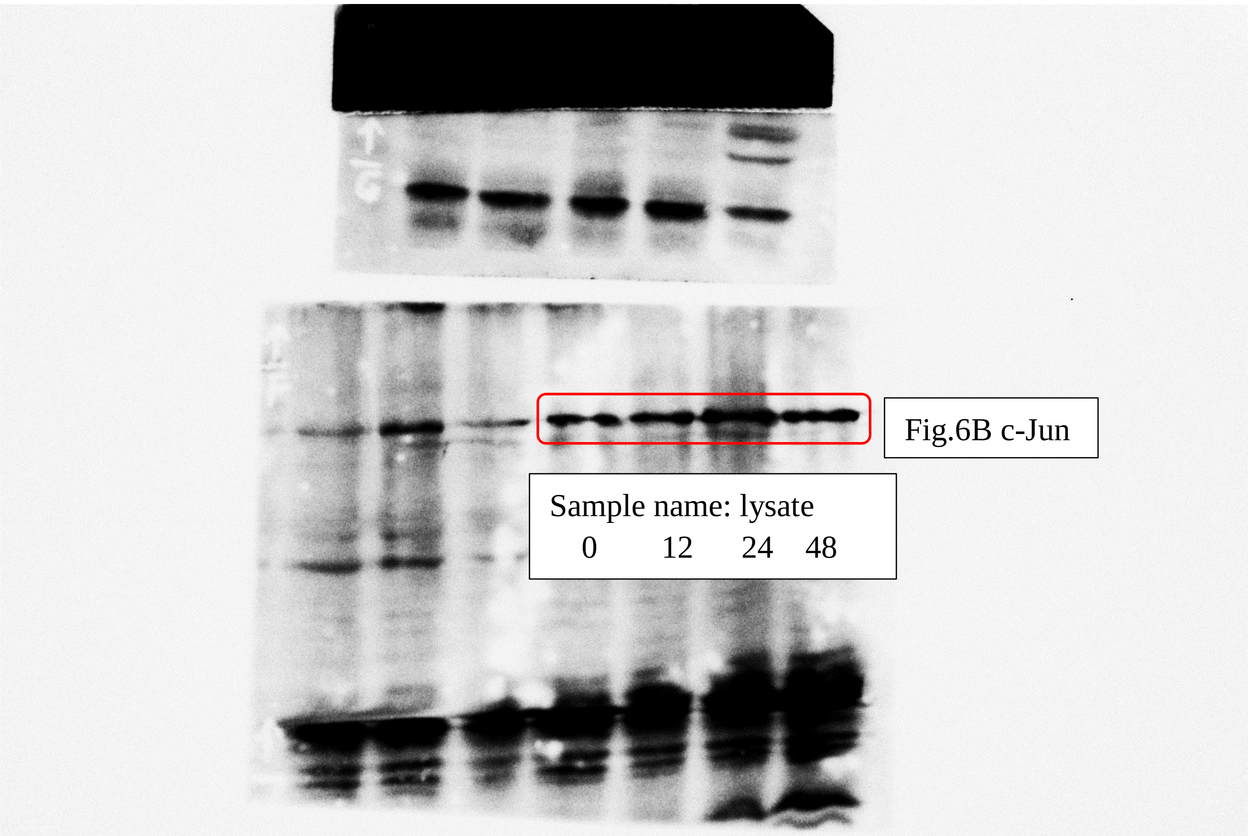
Fig.6B c-Jun
Sample name: lysate
0 12 24 48

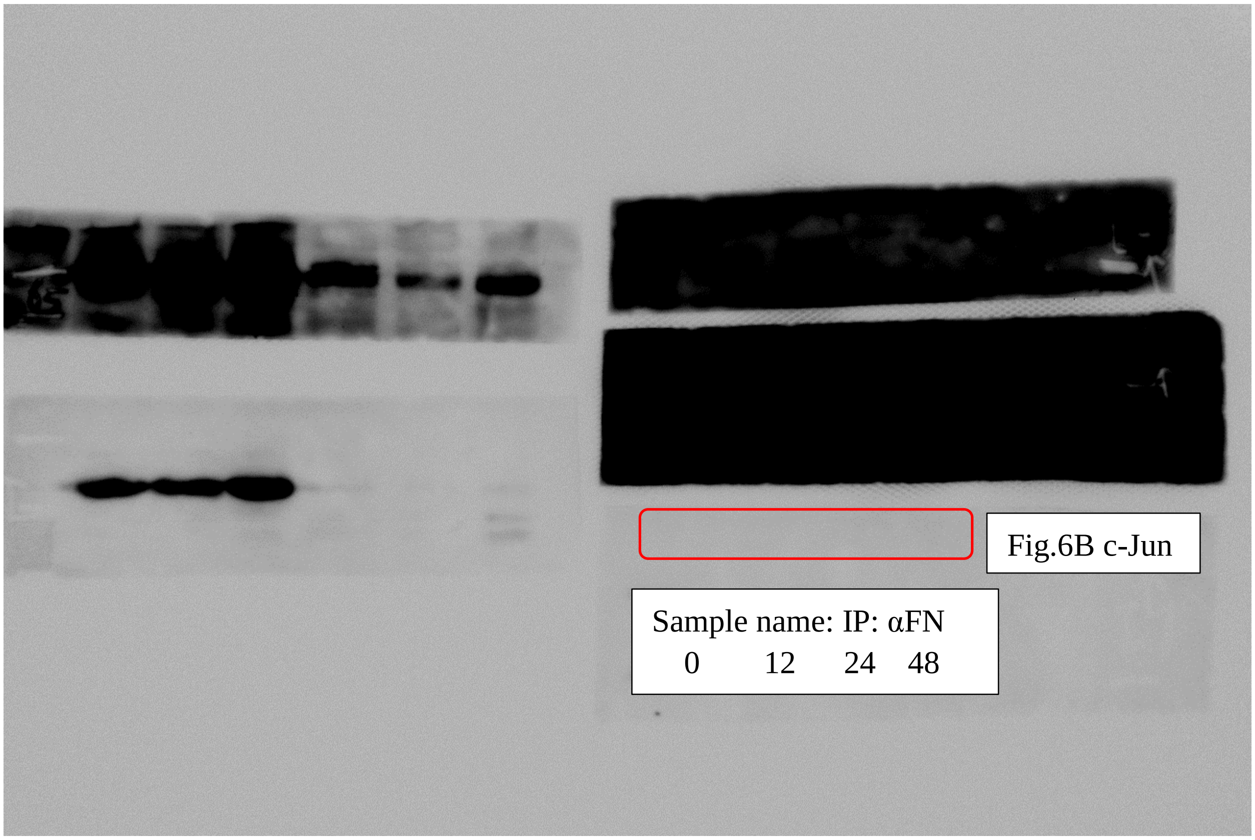
Fig.6B c-Jun
Sample name: IP: αFN
0 12 24 48

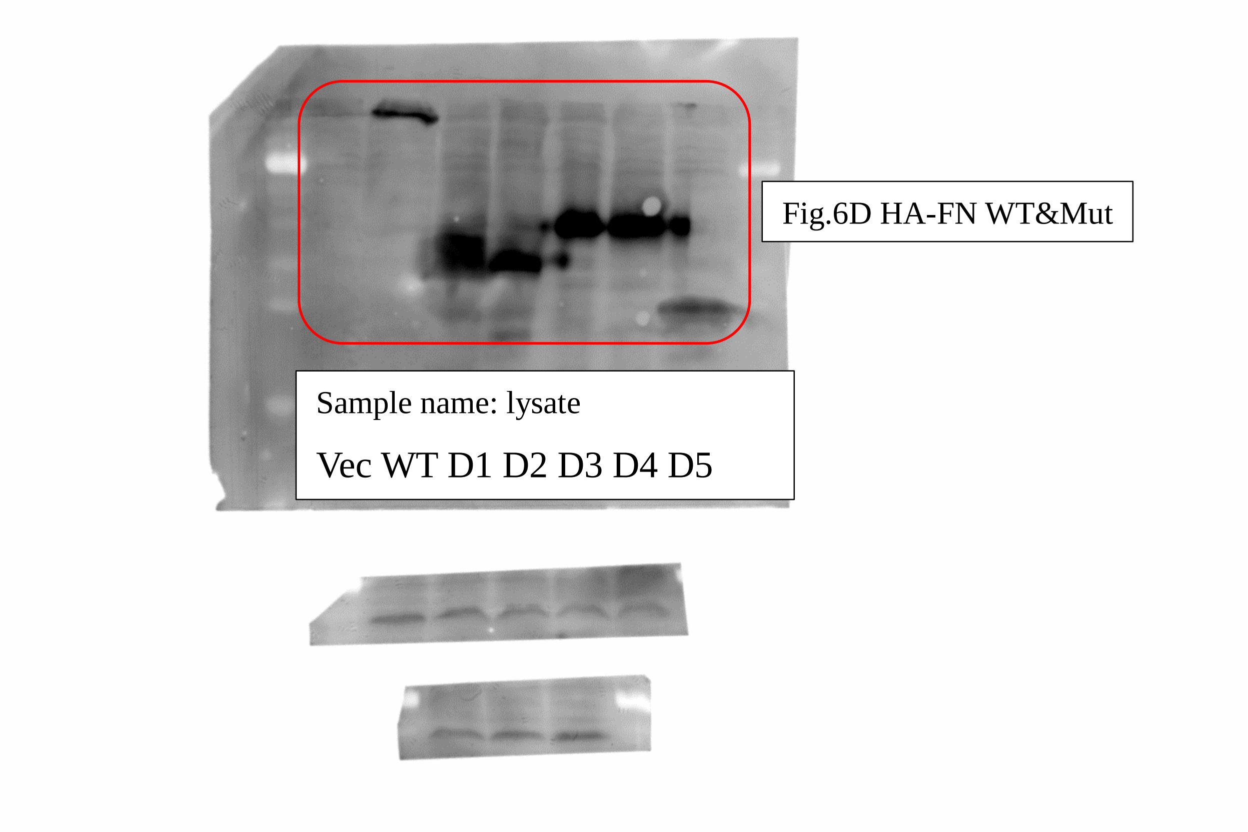
Fig.6D HA-FN WT&Mut
Sample name: lysate
Vec WT D1 D2 D3 D4 D5

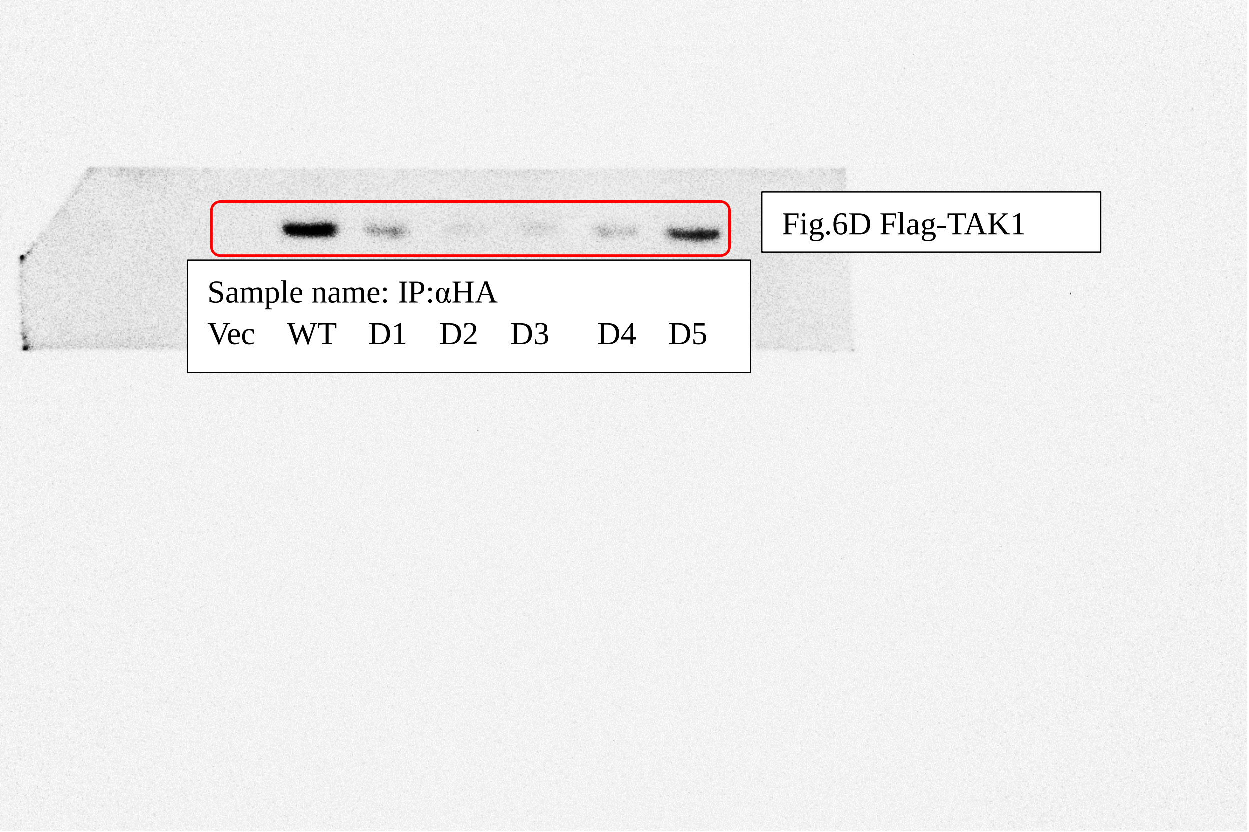
Fig.6D Flag-TAK1
Sample name: IP:αHA
Vec WT D1 D2 D3 D4 D5

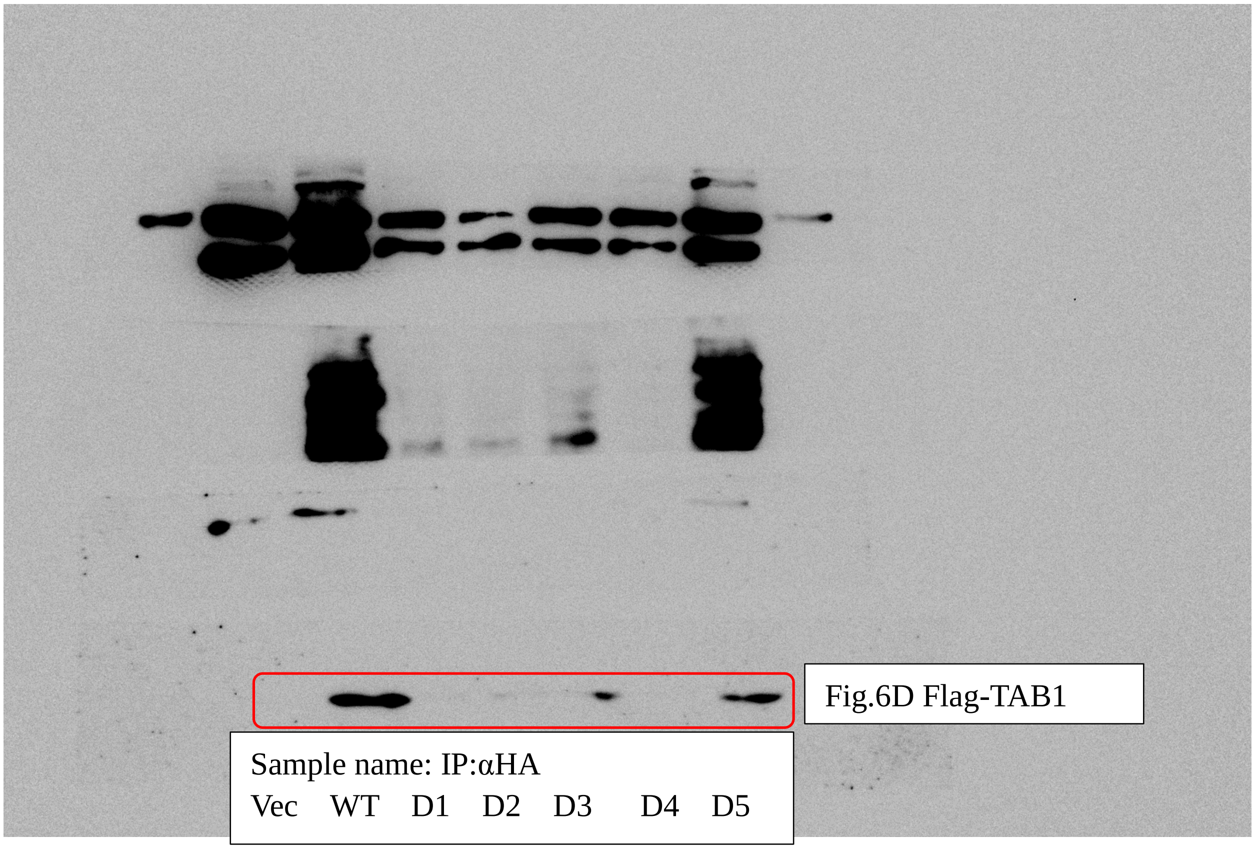
Fig.6D Flag-TAB1
Sample name: IP:αHA
Vec WT D1 D2 D3 D4 D5

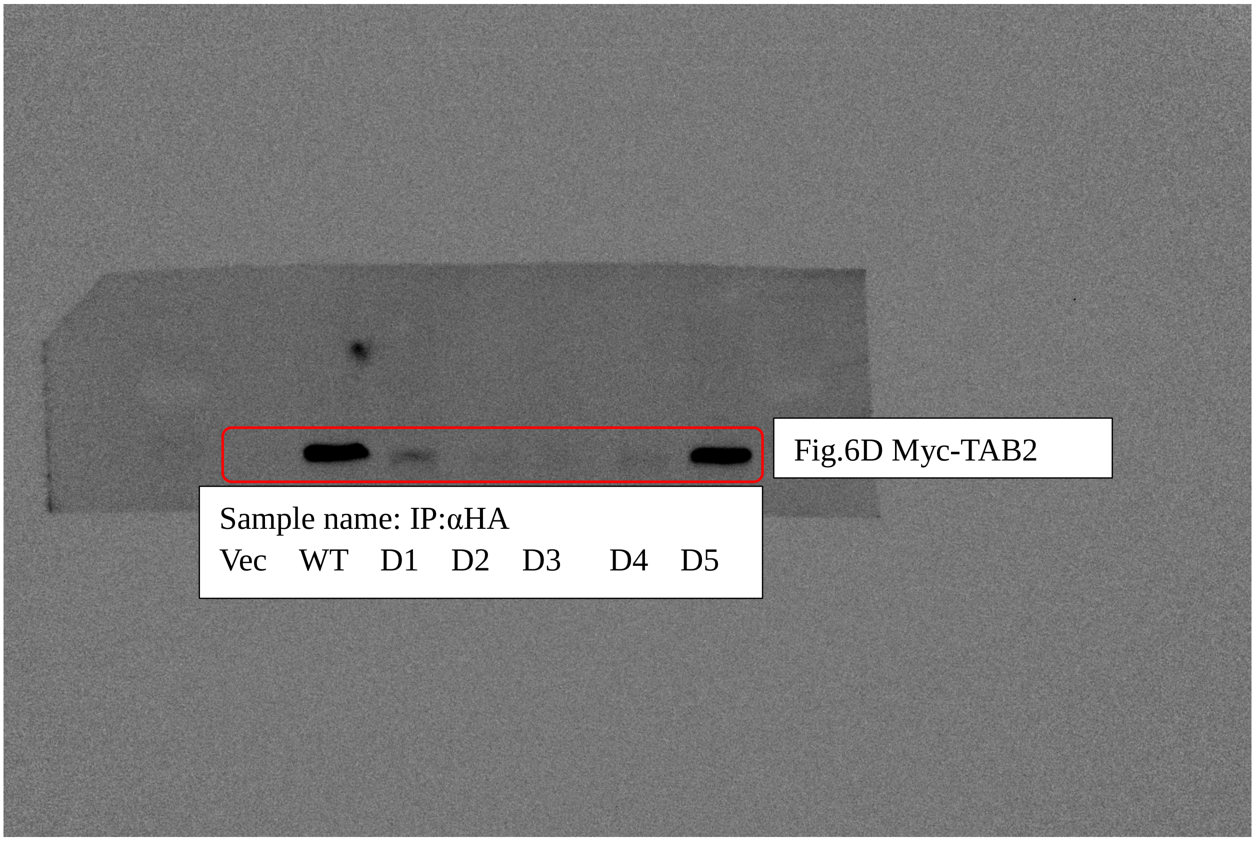
Fig.6D Myc-TAB2
Sample name: IP:αHA
Vec WT D1 D2 D3 D4 D5

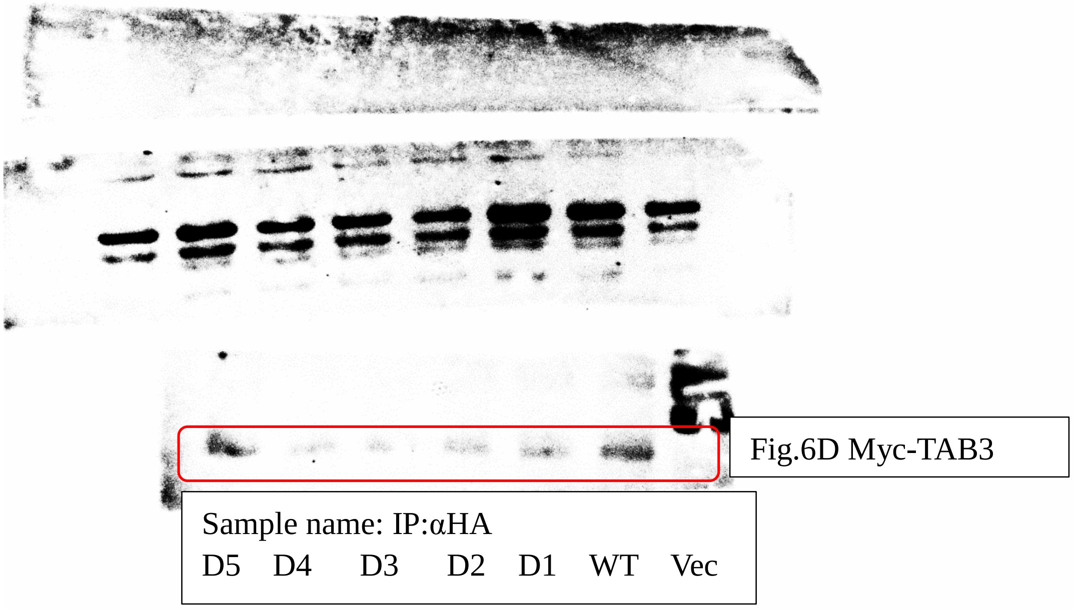
Fig.6D Myc-TAB3
Sample name: IP:αHA
D5 D4 D3 D2 D1 WT Vec

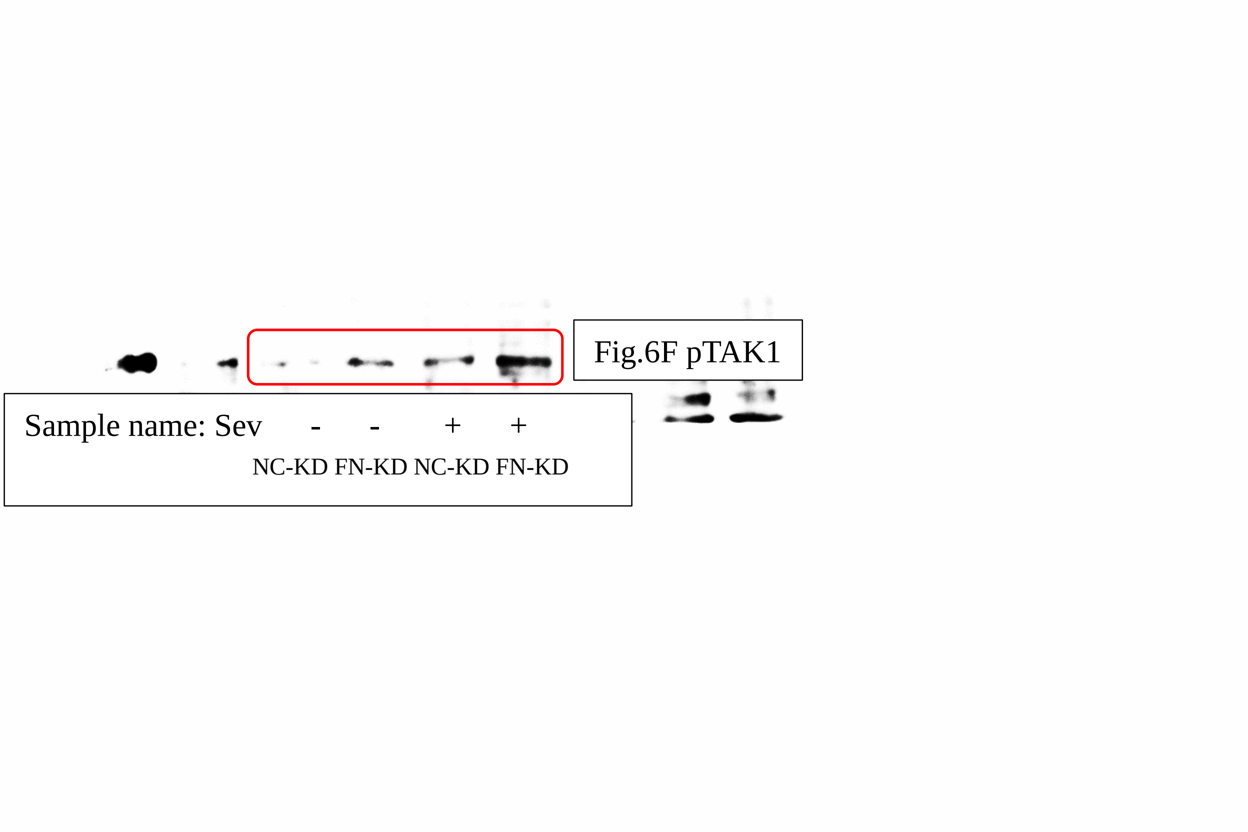
Fig.6F pTAK1
Sample name: Sev - - + +
NC-KD FN-KD NC-KD FN-KD

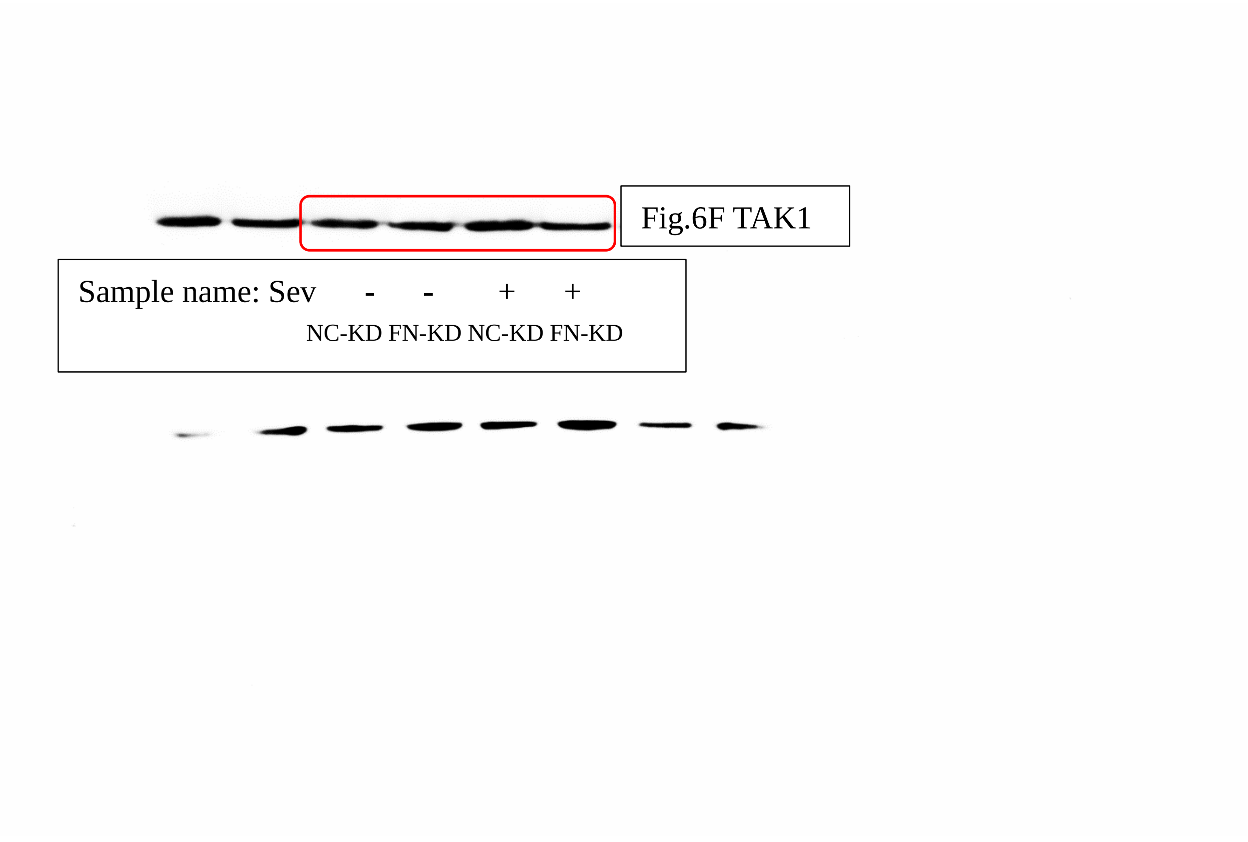
Fig.6F TAK1
Sample name: Sev - - + +
NC-KD FN-KD NC-KD FN-KD

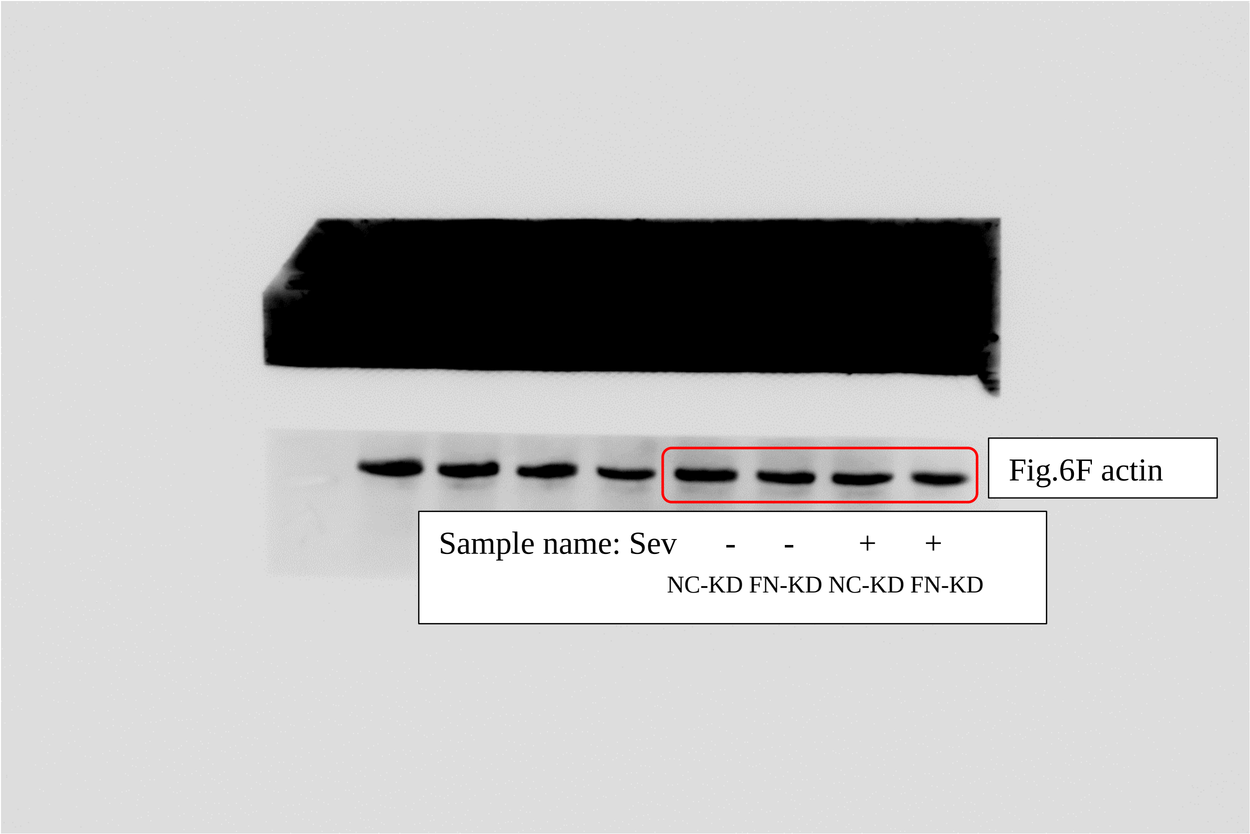

S10 Fig. Original blots in Fig 6.
