# Supplementary material for: Hepatitis B Virus Stimulated Fibronectin Facilitates Viral Maintenance and Replication through Two Distinct Mechanisms
Source: PLoS One. 2016 Mar 29;11(3):e0152721. doi: 10.1371/journal.pone.0152721 (PMC4811540; doi:10.1371/journal.pone.0152721)
Supplement: S11 Fig — (PDF) [file pone.0152721.s011.pdf]

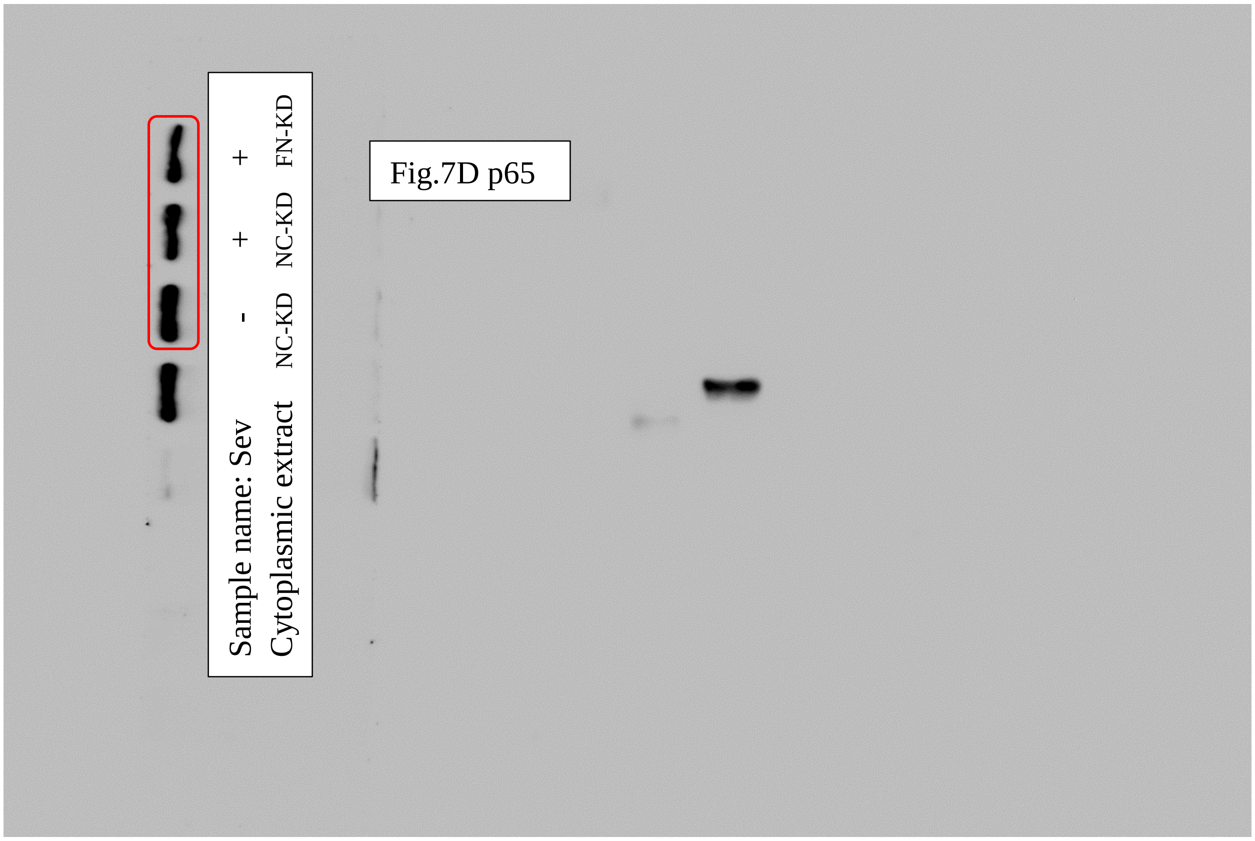
Fig.7D p65
Sample name: Sev
Cytoplasmic extract
-
NC-KD
+
NC-KD
+
FN-KD

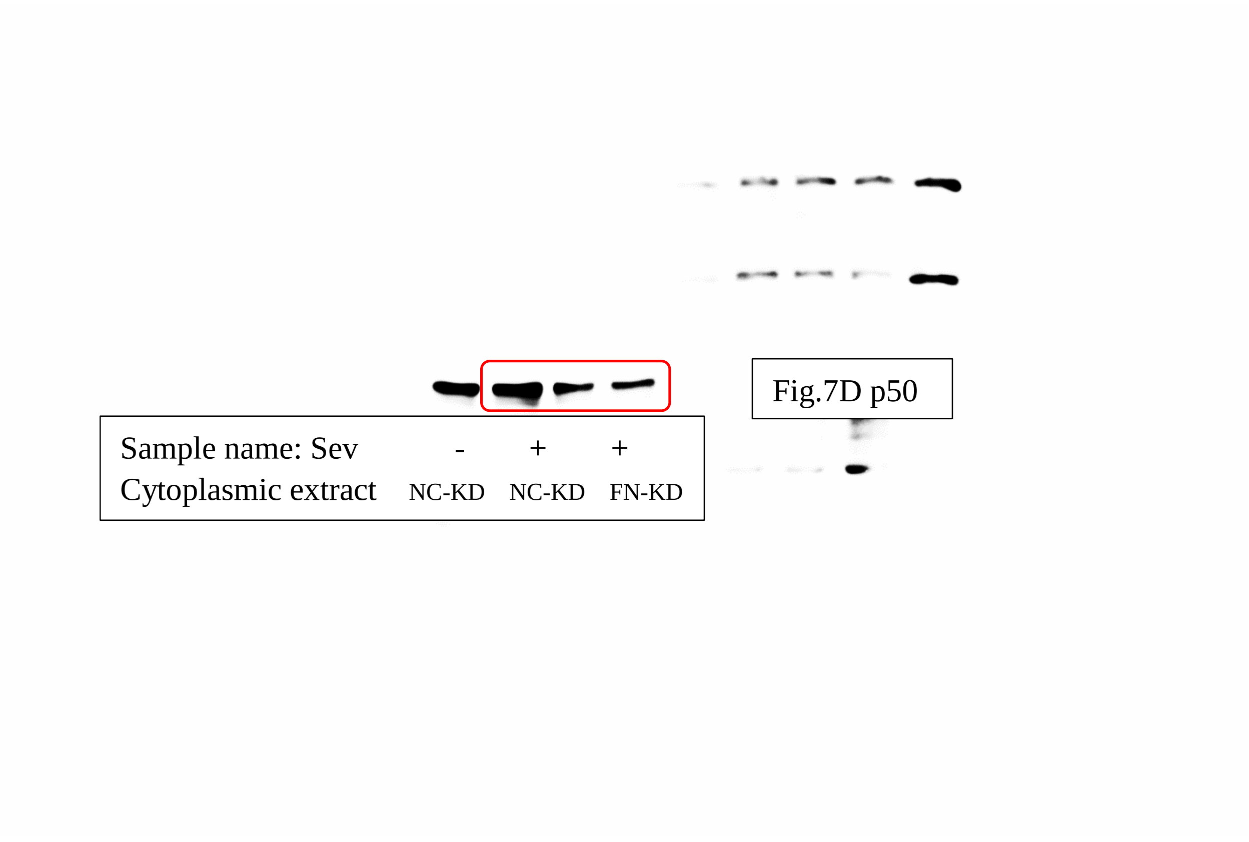
Fig.7D p50
Sample name: Sev
Cytoplasmic extract
-
NC-KD
+
NC-KD
+
FN-KD

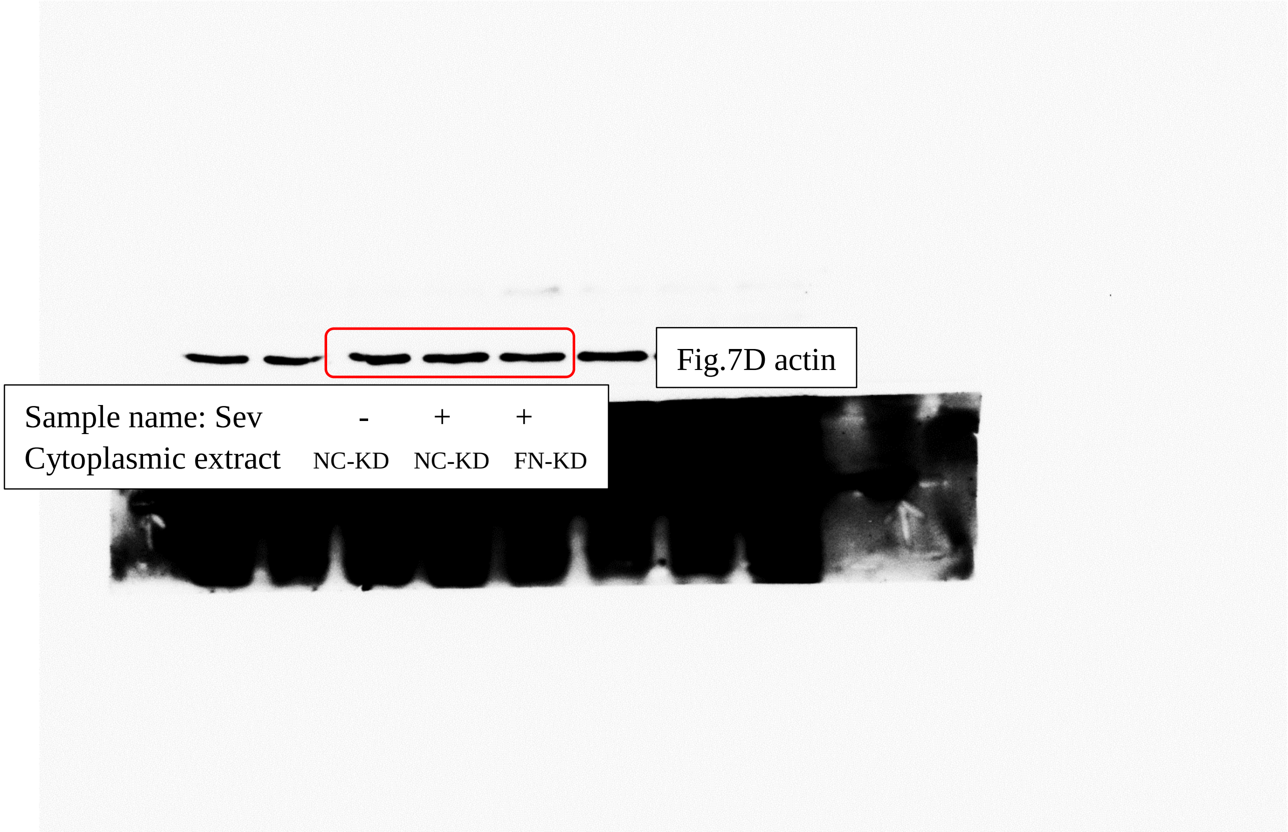
Fig.7D actin
Sample name: Sev - + +
Cytoplasmic extract NC-KD NC-KD FN-KD

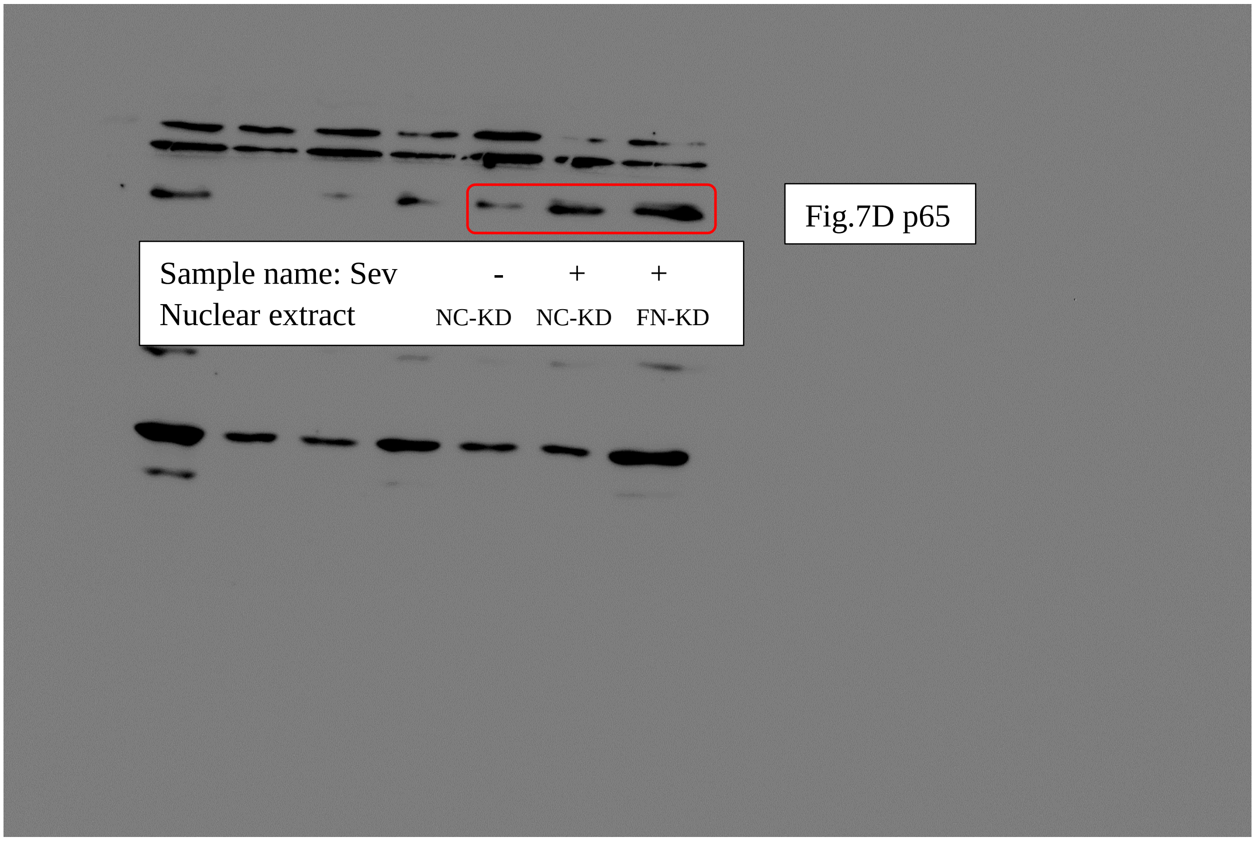
Fig.7D p65
Sample name: Sev - + +
Nuclear extract NC-KD NC-KD FN-KD

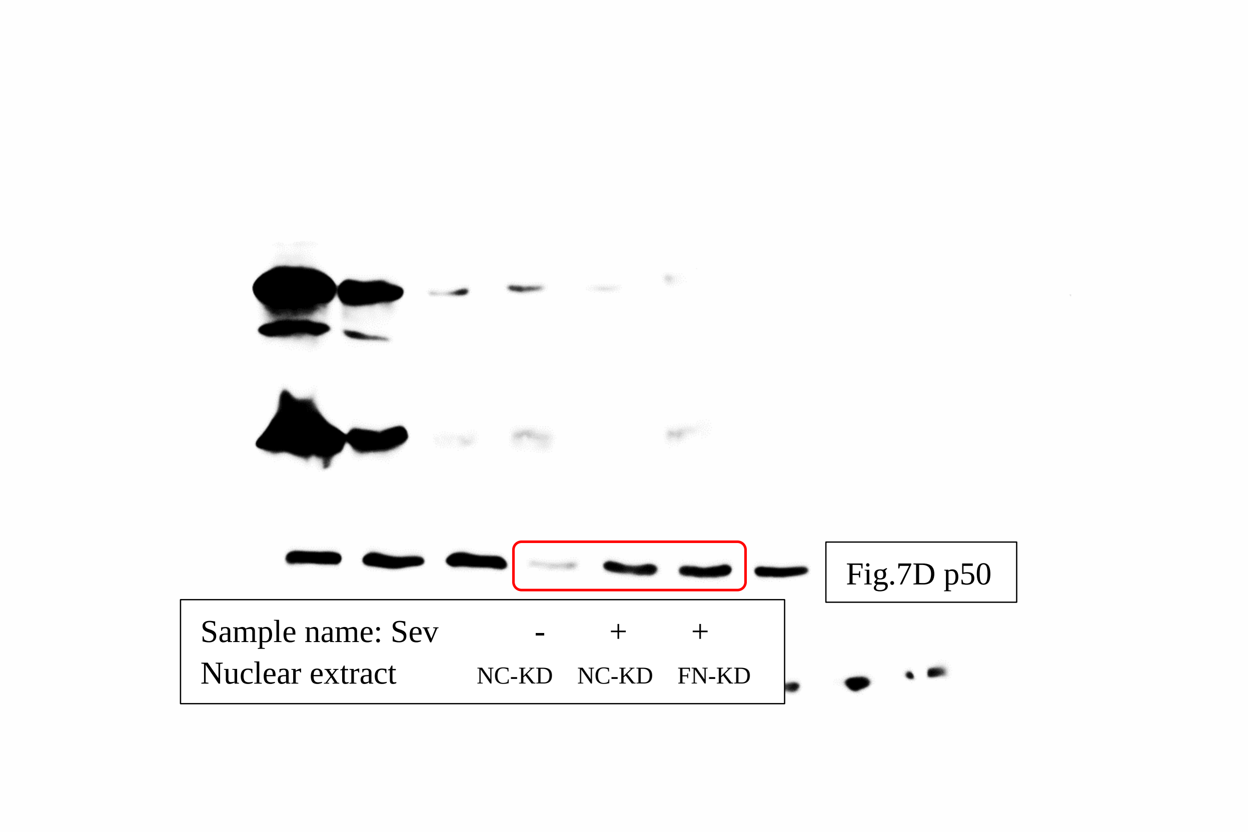
Fig.7D p50
Sample name: Sev - + +
Nuclear extract NC-KD NC-KD FN-KD

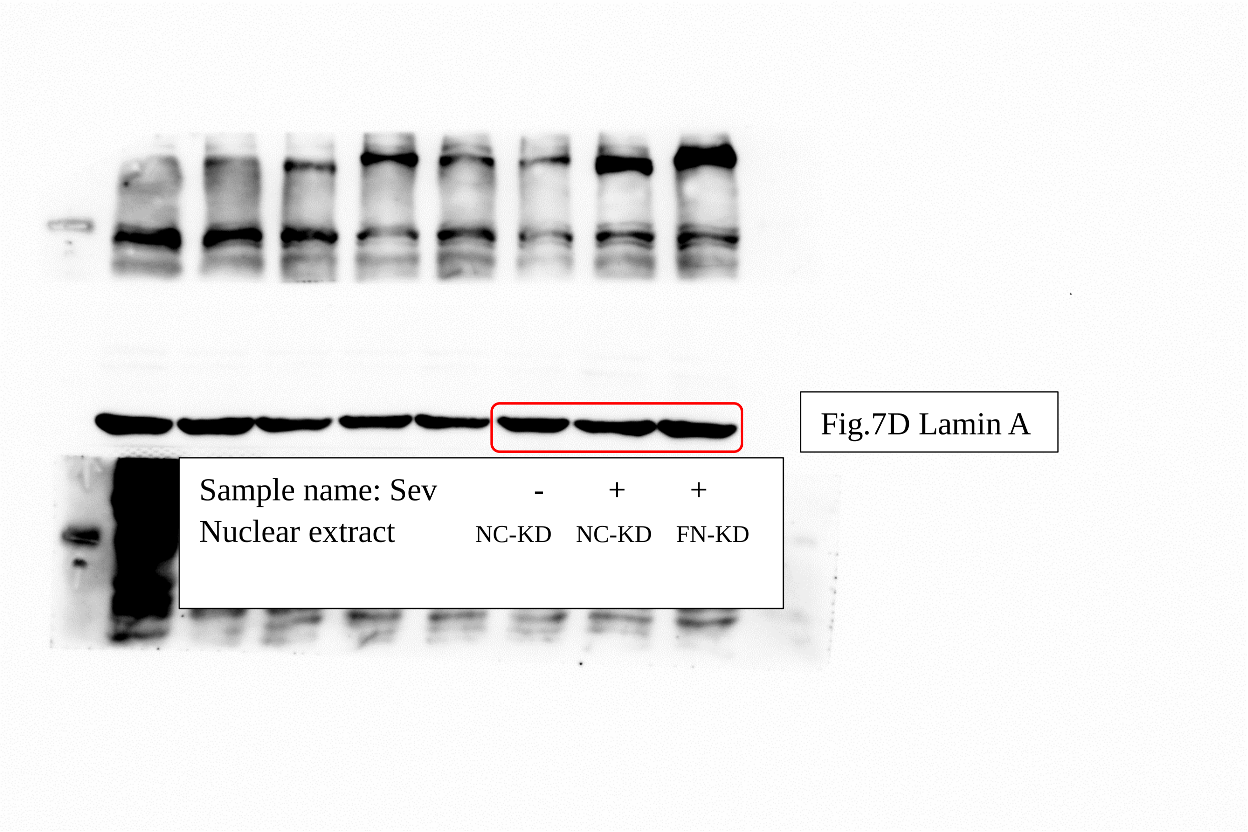
Fig.7D Lamin A
Sample name: Sev - + +
Nuclear extract NC-KD NC-KD FN-KD

S11 Fig. Original blots in Fig 7.
