# Supplementary material for: Hepatitis B Virus Stimulated Fibronectin Facilitates Viral Maintenance and Replication through Two Distinct Mechanisms
Source: PLoS One. 2016 Mar 29;11(3):e0152721. doi: 10.1371/journal.pone.0152721 (PMC4811540; doi:10.1371/journal.pone.0152721)
Supplement: S1 Table — (PDF) [file pone.0152721.s013.pdf]

S1 Table. Baseline Characteristics of HBV-Infected Patients and Healthy individuals in Fig 1A, B.

Characteristic	Healthy individuals(N=50)	HBV Patients(N=50)
Age (years)	44.6±13.3	49.1±15.2
Gender (male/female)	31/19	36/14
HBsAg (+/-)	-	+
HBV genotype (A/B/C/D)	NA	1/26/22/1
WBC (cells/μl)	3032±904	6831±1204
ALT (U/L)	<30	91.16±23.47
HBV DNA (copies/ml)	<500	9.2E + 07±3.5E + 06
FN (μg/ml)	250.2±59.64	531.7±94.75*

Data are presented as mean ± SEM; *p < 0.05 compared with Healthy individuals group; Abbreviations: ALT, alanine aminotransferase.
