# Supplementary material for: Hepatitis B Virus Stimulated Fibronectin Facilitates Viral Maintenance and Replication through Two Distinct Mechanisms
Source: PLoS One. 2016 Mar 29;11(3):e0152721. doi: 10.1371/journal.pone.0152721 (PMC4811540; doi:10.1371/journal.pone.0152721)
Supplement: S2 Table — (PDF) [file pone.0152721.s014.pdf]

S2 Table. Baseline Characteristics of HBV-Infected Patients and Healthy individuals in Fig 1 C.

Characteristic	Healthy individuals(N=15)	HBV Patients(N=15)
Age (years)	49.1±8.39	52.1±12.9
Gender (male/female)	12/3	10/5
HBsAg (+/-)	-	+
HBV genotype (A/B/C/D)	NA	1/6/8/0
WBC (cells/µl)	3318±648	6033±938
ALT (U/L)	<30	95.83±29.39
HBV DNA (copies/ml)	<500	6.8E + 07±4.2E + 06
FN (µg/ml)	263.7±39.57	519.4±75.88*
FN Expression (intensity)		
-	0	0
+	9	3
++	5	5
+++	1	7

For each liver specimen, the antibody-stained sections were carefully examined by two independent observers, and the staining was scored as follows: (-) negative staining, (+) weakly positive staining, (++) moderately positive staining, and (+++) strongly positive staining. The FN staining in uninfected human samples and the FN staining in patients infected with HBV were comparable in intensity.

Data are presented as mean ±SEM; *$P < 0.05$ compared with Healthy individuals group; Abbreviations: ALT, alanine aminotransferase.
