# Supplementary material for: Hepatitis B Virus Stimulated Fibronectin Facilitates Viral Maintenance and Replication through Two Distinct Mechanisms
Source: PLoS One. 2016 Mar 29;11(3):e0152721. doi: 10.1371/journal.pone.0152721 (PMC4811540; doi:10.1371/journal.pone.0152721)
Supplement: S3 Table — (PDF) [file pone.0152721.s015.pdf]

S3 Table. Sequence of shRNA, Q-PCR or RT-PCR primers

sequence name	DNA sequence （5'--3'）	sequence name	DNA sequence （5'--3'）
shNC	GTTCTCCGAACGTGTCACGT	HNF-4α-RT-S	TACGCATCCTTGACGAGCTG
shFN-1	ATAAGTCCTGATACAACCACG	HNF-4α-RT-A	ACTGGCGGTCGTTGATGTAG
shOF-1	ATAAGTCCTCTAACAACCACG	PPARα-RT-S	GCGAACGATTCGACTCAAGC
shFN-2	TATTGATCCCAAACCAAATCT	PPARα-RT-A	CTTGGCATTCGTCCAAAACGA
shOF-2	TATTGATCCGTTACCAAATCT	RXRα-RT-S	ACCGGAACGAGAATGAGGTG
GAPDH-RT-S	AAGGCTGTGGGCAAGG	RXRα-RT-A	TCCACCAGGGTGAAAAGCTG
GAPDH-RT-A	TGGAGGAGTGGGTGTCG	FXRα-RT-S	CGTCAGCAGGGAGGATCAAA
FN-RT-S	CAGGCTCAGCAAATGGTTC	FXRα-RT-A	GCATTCAGCCAACATTCCCA
FN-RT-A	GTAAGTGTTCCCAGTGTACTTGTC	LRH1-RT-S	CGAGTGGGCCAGGAGTAGTA
HBV-RT-S	ATCCTGCTGCTATGCCTCATCTT	LRH1-RT-A	GCTCCGGCTTGTGATGCTAT
HBV-RT-A	ACAGTGGGGAAAGCCCTACGAA	ERRβ-RT-S	TGCGAGATCACCAAACGGAG
HBV probe	TGGCTAGTTTACTAGTGCAATTTTG	ERRβ-RT-A	GCATGGCATAGAGCTTGTCC
IFN-α-RT-S	TTTCTCCTGCCTGAAGGACAG	ERRγ-RT-S	CGATGCCCAAGAGACTGTGT
IFN-α-RT-A	GCTCATGATTTCTGCTCTGACA	ERRγ-RT-A	GTCAAGACGCACCCCCTTCTT
OAS2-RT-S	ACCTTGGAAAGTGCCGACAA	chip-EI-RT-S	CCTGCGTTAATGCCCTTGTA
OAS2-RT-A	CAAGTTGACAGAAATAAGATGCACA	chip-EI-RT-A	GGGTTGCGTCAGCAAACAC
MxA-RT-S	GCCGGCTGTGGATATGCTA	chip-EII-RT-S	TGAACGCCCACCGAATGTT
MxA-RT-A	TTTATCGAAACATCTGTGAAAGCAA	chip-EII-RT-A	CTACAGCCTCCTAGTACAAAGACC
PKR-RT-S	AGAGTAACCGTTGGTGACATAACCT	FN-sp1-chip-S	TCTTGCAACCCCTTCGCTTC
PKR-RT-A	GCAGCCTCTGCAGCTCTATGTT	FN-sp1-chip-A	CGGGGCTTATATGGGACGGT
AP1-WT（-428... -421）	TGACGCA	ATF2-WT (-184… -175)	GTGACGTCAC
AP1-Mut	TGTTTTA	ATF2-Mut	GTTTTTGCAC
AP2-WT (-299... -292)	CCCCATCC	SP1-WT (-107… -100)	GGGCGGGC
AP2-Mut	CCGGGGC	SP1-Mut	GGAAAAGC
PEA2-WT (-275... -269)	GACCGCA	AP2-WT (-67… -60)	CCCCGGGC
PEA2-Mut	GATTTTA	AP2-Mut	CCTTTTGC
AP2-WT (-243… -235)	CCCCAGTC	SP1-WT (-49… -44)	GGGCGG
AP2-Mut	CCTTTTTC	SP1-Mut	GTTTTG
EI-WT(1134… 1142)	TGAACCTTTA	EII-WT (1664… 1673)	TGAGAACCTG
EI-Mut	TCTACGGCTA	EII-Mut	TGTTTTGCTG
siNC	GTTCCGACGTTTCACGTACGAG	si-IFNAR1	CCTTAGTGATTCATTCCATAT
siFN	GGAGATGAGTGGGAACGAATG		
